# Overcoming Transmission Barriers: A Dual‐Functional Adhesive Hydrogel for Direct Monkeypox Virus Neutralization and Wound Healing

**DOI:** 10.1002/advs.76926

**Published:** 2026-08-05

**Authors:** Ming Zhang, Jiaxin Tian, Xiaolong Zhu, Xiao Li, Xiangshu Qiu, Yuanyuan Li, Ben Zhong Tang, Ning Shi

**Affiliations:** ^1^ State Key Laboratory Cultivation Base of Research The Affiliated Stomatological Hospital of Nanjing Medical University Prevention and Treatment for Oral Diseases Jiangsu Province Engineering Research Center of Stomatological Translational Medicine Nanjing Medical University Nanjing China; ^2^ State Key Laboratory For Diagnosis and Treatment of Severe Zoonotic Infectious Diseases Institute of Zoonosis and College of Veterinary Medicine Key Laboratory for Zoonosis Research of the Ministry of Education Jilin University Changchun China; ^3^ Changchun Veterinary Research Institute Chinese Academy of Agricultural Sciences Changchun China; ^4^ Guangdong Basic Research Center of Excellence for Aggregate Science School of Science and Engineering The Chinese University of Hong Kong (Shenzhen) Shenzhen Guangdong China

**Keywords:** antioxidant, conjugated polymer, inflammation regulation, mesoporous polydopamine, monkeypox

## Abstract

The local inflammatory response and ongoing viral replication caused by monkeypox virus (MPXV) infection pose severe challenges for clinical treatment. Traditional treatment strategies are unable to achieve the dual goals of virus clearance and tissue repair simultaneously. This study innovatively adopts a hierarchical assembly strategy of “mesoporous carrier anchoring‐gel matrix embedding” to construct a functionalized hydrogel (Polymer@mPDA@Gel). Using mesoporous polydopamine (mPDA) as the exclusive anchoring site to load the polymer, forming an efficient photothermal component, and then embedding this component in a gel matrix with strong adhesion and good biocompatibility. Through hierarchical assembly, the photothermal performance, anti‐inflammatory performance, and biointerfacial properties are precisely integrated to lay the core foundation for its anti‐viral and anti‐inflammatory functions. The functionalized hydrogel constructed realizes targeted clearance of MPXV, regulation of inflammation, and integration of tissue repair, providing a new material technology path and theoretical support for the clinical treatment of MPXV infection.

## Graphical abstract

MPXV infection leads to persistent viral replication and severe local inflammation, while existing treatments cannot simultaneously remove viruses and repair injured tissue. This study constructs Polymer@mPDA@Gel hydrogel via a two‐step hierarchical assembly method: mPDA anchors photothermal polymers to form antiviral photothermal subunits, which are further embedded into biocompatible sticky hydrogel. This structural design integrates photothermal, anti‐inflammatory and bio‐interfacial advantages. The material realizes targeted MPXV clearance, inflammatory homeostasis regulation and tissue regeneration all‐in‐one, establishing new material strategies and theoretical guidance for clinical monkeypox treatment.

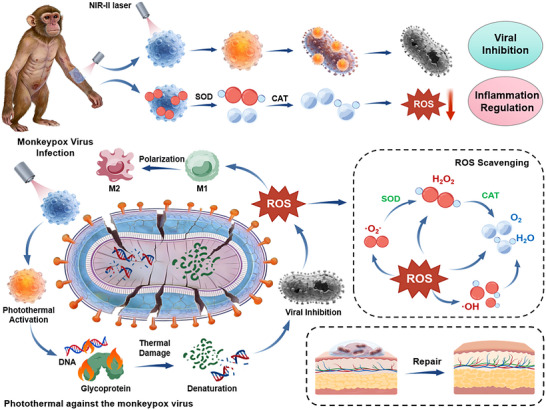

## 1 Introduction

Since its initial identification in monkeys at a Danish laboratory in 1958, the monkeypox virus (MPXV) has progressively expanded its epidemiological footprint [1, 2, 3]. Early occurrences were largely confined to sporadic outbreaks in several Central and West African countries, where geographic isolation, limited transmission routes, and diagnostic challenges constrained its spread, rendering it a regional public health concern. However, accelerated globalization, increased human mobility, and ecological changes have facilitated the virus's transboundary dissemination [4, 5]. The global burden remains substantial, with 106,310 laboratory‐confirmed cases reported across 123 countries and regions, including 234 fatalities, and case numbers continue to increase [6]. Currently, the clinical management of MPXV infection primarily involves supportive care along with antiviral therapy [7]. Standard care involves debridement of these lesions, along with analgesia and antibiotics to prevent secondary bacterial infections. Concurrently, antiviral agents such as tecovirimat and brincidofovir, both approved by the U.S. Food and Drug Administration (FDA) for the treatment of smallpox, are administered to reduce viral load [8, 9]. However, these drugs exhibit notable limitations; although tecovirimat is currently the first‐line therapeutic option for monkeypox, several cases of potential tecovirimat resistance have been reported worldwide [10]. Brincidofovir, on the other hand, is associated with significant toxicity and suboptimal efficacy, thus serving only as an alternative when tecovirimat is unavailable or contraindicated [11]. Therefore, developing a unique strategy to target and eradicate the monkeypox virus in skin and mucous membranes, thereby blocking viral transmission at its source, while achieving precise treatment of monkeypox, holds significant clinical importance.

Photothermal therapy (PTT) is a novel treatment method that uses photosensitizers to convert light energy into heat under specific‐wavelength light irradiation, enabling targeted destruction of diseased tissues through localized hyperthermia [12, 13, 14, 15, 16]. Compared to traditional antiviral drugs, PTT offers benefits such as high targeting accuracy, a low risk of inducing drug resistance, and minimal toxicity [17, 18]. As a type of photosensitizer, cationic conjugated polymers can effectively overcome the main limitation of conventional photosensitizers, which often experience fluorescence quenching due to π‐π stacking during aggregation, while also displaying excellent optical properties [19, 20, 21, 22]. In the aggregated state, the intramolecular rotation and vibration of cationic conjugated polymer photosensitizers are restricted; when they absorb near‐infrared light, the energy is efficiently converted into heat rather than being lost through non‐radiative transitions [23, 24]. The locally increased temperature can directly disrupt the viral protein shell, such as the lipid envelope of enveloped viruses or the capsid proteins of non‐enveloped viruses, leading to structural denaturation [25, 26]. Additionally, heat can damage the secondary and tertiary structures of viral nucleic acids (DNA/RNA), making the virus unable to replicate or infect [27, 28]. Although cationic conjugated polymers, with their efficient photothermal conversion mechanism, can break down viral structures and eliminate infected cells through precise local heat generation—offering a powerful tool for treating viral infections—it's important to remember that viral infections themselves trigger immune responses, resulting in a large release of inflammatory factors (e.g., TNF‐α, IFN‐γ) at the infection site [29, 30, 31, 32, 33]. If the inflammatory response becomes uncontrolled, excessive cytokine release can worsen local tissue oxidative stress and cause cellular damage [34]. This not only risks enlarging ulcerated areas and intensifying tissue necrosis but may also disrupt the necessary “inflammation–repair” balance for wound healing, thereby delaying epithelial cell regeneration and granulation tissue formation, ultimately impeding recovery after treatment [35, 36].

Polydopamine (PDA) has demonstrated outstanding performance in drug delivery and inflammation regulation owing to its unique chemical structure and excellent biocompatibility [37, 38]. Functional groups in its molecular structure, such as catechol and amino groups, enable the loading of various therapeutic agents, including small‐molecule drugs and biomacromolecules, through hydrogen bonding, π‐π stacking, and electrostatic interactions [39, 40]. Meanwhile, PDA can directly scavenge excess reactive oxygen species (ROS) in the inflammatory microenvironment through its catechol groups and inhibit peroxidase activity, thereby reducing ROS generation [41]. It also suppresses inflammatory signaling pathways, decreasing the secretion of pro‐inflammatory cytokines such as TNF‐α and IFN‐g, while promoting the expression of anti‐inflammatory factors such as IL‐10 and TGF‐β, thereby reprogramming the inflammatory microenvironment [37]. Additionally, PDA facilitates fibroblast proliferation and epithelial cell migration, thereby accelerating the repair of inflammation‐damaged tissues [42]. As wound dressings, hydrogels play a critical role in wound healing due to their high water content, favorable biocompatibility, and versatile functional adaptability [43, 44, 45]. Their strong water absorption and retention capabilities allow them to absorb wound exudate and maintain a moist wound environment, providing optimal conditions for cell proliferation and migration [43]. Furthermore, some functionalized hydrogels enable the controlled release of active ingredients such as drugs and growth factors, offering targeted inhibition of infection and promoting tissue regeneration [46]. Their soft and adaptable texture conforms well to irregular wound shapes, reduces mechanical stress on newly formed tissues during dressing changes, and improves patient comfort [47]. These properties make hydrogels suitable for a wide range of wound types, including abrasions, burns, and chronic ulcers [43].

This study proposed an innovative dual‐assembly strategy, termed “mesoporous carrier anchoring‐gel matrix embedding”, to construct a multifunctional hydrogel integrating photothermal effects and immunomodulatory functions. Based on this functionalized hydrogel, a novel integrated therapy, termed “virus inhibition + inflammation regulation”, is proposed for MPXV. This functionalized material achieves the precise integration of three key functions: (1) The efficient photothermal conversion property of Polymer@mPDA generates localized hyperthermia for targeted photothermal viral eradication. (2) The inherent antioxidant activity of the mPDA carrier effectively scavenges excess reactive oxygen species (ROS) at the infection site and modulates the balance of inflammatory factors, enabling precise regulation of the inflammatory microenvironment. (3) The high adhesion of the gel matrix ensures close contact with the infected lesion, facilitating the synergistic action of photothermal sterilization, ROS scavenging, and inflammation modulation. Based on this functionalized hydrogel, a novel integrated therapy—termed “virus inhibition + inflammation regulation”—is proposed for MPXV. This strategy leverages the superior photothermal conversion and antioxidant properties of Polymer@mPDA to achieve targeted MPXV clearance at the infection site, inhibit viral replication, regulate the inflammatory microenvironment, and accelerate tissue repair. Consequently, this approach accomplishes the dual therapeutic goals of antiviral activity and tissue regeneration, providing a novel material system to support precision therapy for MPXV infection.

## 2 Results

### 2.1 Preparation and Characterization of Polymer@mPDA

**FIGURE 1:**
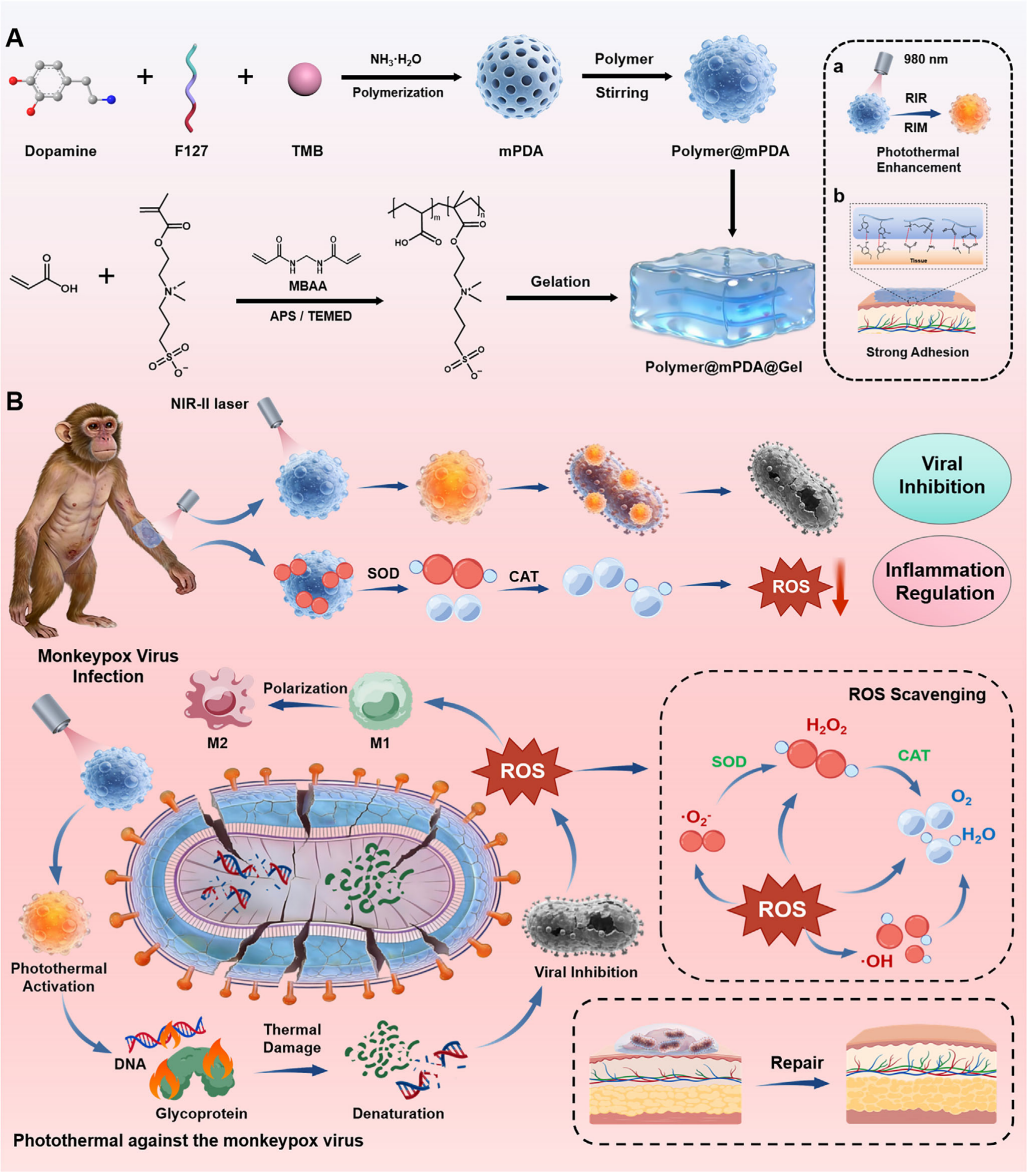
Schematic illustration of the synthesis method and therapeutic strategy of Polymer@mPDA@Gel. (A) Scheme diagram of the dual‐loading strategy‐based hydrogel Polymer@mPDA@Gel and (B) therapeutic strategy featuring “viral inhibition + inflammation regulation” against MPXV.

**FIGURE 2:**
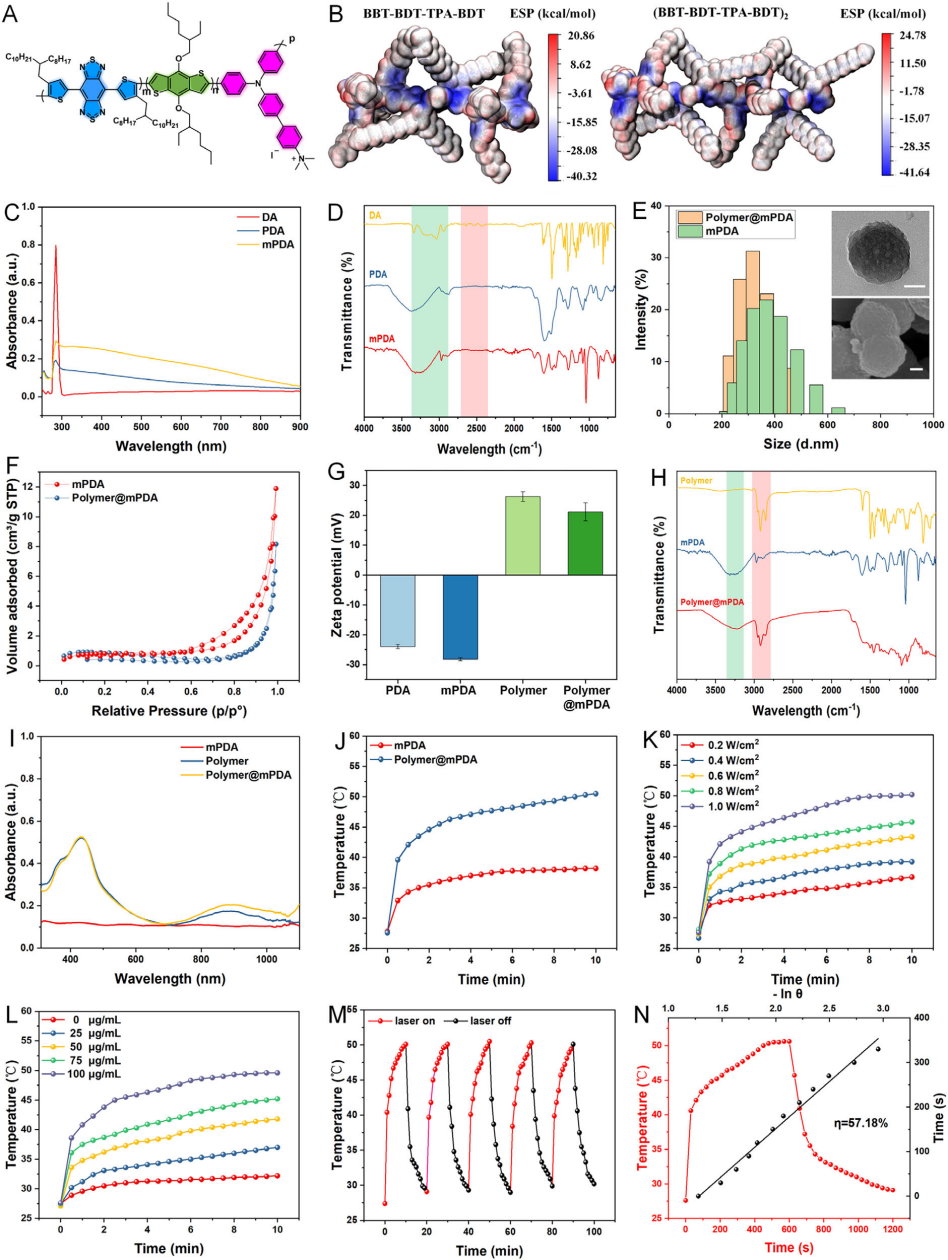
Characterization and photothermal performance of mPDA. (A) The structural formula of a polymer. (B) ESP maps of different repeating units. (C) UV–vis absorption spectra and (D) FTIR spectrum of DA, PDA, and mPDA. (E) Particle size histograms of mPDA and Polymer@mPDA, as well as TEM images of mPDA and SEM images of Polymer@mPDA. Scale bar, 200 nm. (F) Nitrogen sorption isotherms of mPDA and Polymer@mPDA. (G) Zeta potential of PDA, mPDA, Polymer, and Polymer@mPDA. (H) FTIR spectrum of Polymer, mPDA, and Polymer@mPDA. (I) UV–vis absorption spectra of mPDA, Polymer, and Polymer@mPDA. (J) The heating curves of mPDA and Polymer@mPDA under irradiation with a 980 nm laser at a power density of 1.0 W/cm^2^. Heating curves of Polymer@mPDA under irradiation with a 980 nm laser at (K) different power densities and (L) at different concentrations. (M) Heating and cooling curves of Polymer@mPDA over five on‐off laser cycles. (N) Calculation of the photothermal conversion efficiency of Polymer@mPDA under 980 nm laser irradiation (1.0 W/cm^2^). For G, data are represented as the mean ± s.d. (*n* ≥ 3). One‐way analysis of variance (ANOVA) with post‐hoc corrections was applied. Experiments in C–F and H–M were repeated independently three times with consistent results, and a representative result is shown for each.

In this study, we proposed an innovative dual‐assembly strategy, termed “mesoporous carrier anchoring‐gel matrix embedding”, to construct a multifunctional hydrogel integrating photothermal effects and immunomodulatory functions. The core design is as follows: mesoporous polydopamine (mPDA) nanoparticles were synthesized using Pluronic F127 as a soft template, 1,3,5‐trimethylbenzene (TMB) as a porogen, and dopamine (DA) as the carbon/nitrogen source. These mPDA nanoparticles served as specific loading sites to anchor the polymer, forming an efficient photothermal agent (Polymer@mPDA) (Figure 1). Subsequently, a gel matrix with high adhesion and favorable biocompatibility was prepared through the copolymerization of acrylic acid (AA) and sulfobetaine methacrylate (SBMA). The photothermal agent was then embedded within this gel system, ultimately constructing the multifunctional composite material (Polymer@mPDA@Gel). The conjugated polymers were constructed using benzodithiophene (BDT) and benzodithiadiazole (BBT) derivatives as donors and acceptors, respectively, in which the intermediate thiophene ring was functionalized to *π*‐conjugated groups. Cationic polymers were obtained by protonation (Figure S1), the structural formula of the polymer is shown in Figure 2A. The energy level difference between the highest occupied molecular orbital and the lowest unoccupied molecular orbital (HOMO–LUMO) in various repeating units essentially reflects the energy required for electrons to transition from the HOMO to the LUMO within the material, which directly determines its key optical and electrical properties. By analyzing the HOMO and LUMO distributions, the electronic structures of repeating units composed of different monomer units (BBT, BDT, and TPA) and of the target repeating units were systematically investigated. The HOMO–LUMO gaps of the monomer and dimer are 1.28 eV for BBT‐BDT‐TPA‐BDT and 1.21 eV for (BBT‐BDT‐TPA‐BDT)_2_, respectively. Meanwhile, the electrostatic potential (ESP) maps of different repeating units reveal significant differences in charge‐distribution polarity between the molecules (Figure 2B). These characteristics suggest that significant intramolecular charge separation can occur under photoexcitation.

Porous‐free PDA and mPDA nanoparticles were prepared via oxidative self‐polymerization and soft‐template methods, respectively [48]. Due to the oxidation of dopamine to form dopachrome and dopamine, followed by self‐polymerization, PDA and mPDA exhibit significant absorption characteristics across the ultraviolet to near‐infrared range in ultraviolet–visible spectroscopy (UV–vis), which distinctly differ from those of dopamine monomers. To investigate their optical properties, we conducted relevant studies, and the results are shown in Figure 2C. Dopamine monomers contain a benzene ring, displaying an absorption peak at 280 nm corresponding to the B‐band of the benzene ring. In contrast, polydopamine, formed through the self‐polymerization of dopamine monomers, develops more conjugated structures, such as quinone‐like configurations, during polymerization. These new conjugated structures enhance the electron delocalization in polydopamine, reduce the energy gap between molecular orbitals, and cause a redshift in the absorption wavelength. Consequently, the absorption peak intensity of polydopamine at 280 nm is significantly weakened, and its absorption spectrum extends into the near‐infrared region.

The differences in the chemical composition of DA, PDA, and mPDA were further analyzed through Fourier transform infrared (FTIR) spectroscopy experiments, with the results shown in Figure 2D. For DA, the broad peak observed at approximately 3000–3400 cm^−1^ in its FTIR spectrum can be attributed to intermolecular hydrogen bonding, while the peaks at 2433.75, 2539.81, 2640.09, and 2750.52 cm^−1^ originate from the stretching vibrations of aromatic NH_2_. After polymerization, these four peaks in the 2433.75–2750.52 cm^−1^ range disappeared, likely due to the conversion of NH_2_ to secondary amines during the process. The broad peak at 3200–3600 cm^−1^ became wider compared to that of DA, which is associated with the stretching vibrations of phenolic hydroxyl (O─H) and amino (N─H) bonds related to catechol groups during the synthesis of PDA. PDA NPs with intramolecular/intermolecular cross‐linking were formed through spontaneous oxidative polymerization of monomers, involving non‐covalent interactions such as hydrogen bonding, *π*–*π* stacking, hydrophobic effects, and charge transfer. Furthermore, no significant differences were observed between PDA and mPDA, indicating that they are different forms of the same substance. The FTIR experimental results confirm the successful synthesis of PDA and mPDA.

Subsequently, the conjugated polymer was grafted onto mPDA to prepare polymer@mPDA. The microscopic morphology and size of PDA, mPDA, and polymer@mPDA were characterized using scanning electron microscope (SEM), transmission electron microscopy (TEM), and dynamic light scattering (DLS), as shown in Figure S2. The SEM images reveal that PDA presents as uniform spheres with a smooth surface and a diameter of 441.2 (±16.98) nm. To observe the mesoporous structure of mPDA, TEM was employed for characterization. The images clearly show distinct mesoporous channels in mPDA. Meanwhile, the SEM images of polymer@mPDA indicate a relatively rough surface with noticeable uneven morphology, indicating that the polymer was successfully grafted onto the surface of mPDA. The diameter distribution histograms of mPDA and polymer@mPDA largely overlap (Figure 2E).

The mesoporous characteristics of mPDA and Polymer@mPDA were investigated using N_2_ adsorption‐desorption experiments to compare their pore structures. The specific surface area of mPDA was measured to be 3.79 m^2^/g. In comparison, that of Polymer@mPDA was 2.98 m^2^/g (Figure 2F). This decrease in specific surface area after polymer loading indicates that the polymer partially filled the pores of mPDA, resulting in reduced accessibility of the mesoporous structure. Subsequently, the zeta potentials of the respective materials were measured. The results indicated that both PDA and mPDA exhibited negative surface charges, with values of −23.93 and −28.2 mV, respectively. In contrast, the polymer showed a positive surface charge of 26.31 mV. After grafting with the polymer, the zeta potential of Polymer@mPDA was recorded as 21.13 mV (Figure 2G). The reversal of surface charge from negative to positive provides strong evidence for the successful grafting of the polymer onto the mPDA surface. Furthermore, FTIR was performed on the polymer, mPDA, and Polymer@mPDA samples, as shown in Figure 2H, mPDA displayed a broad absorption peak between 3200 and 3600 cm^−1^, which is attributed to the presence of numerous phenolic hydroxyl groups. In comparison, the polymer exhibited a relatively weak absorption in this region but showed distinct characteristic peaks between 2800 and 3000 cm^−1^, corresponding to the C─H stretching vibrations of aliphatic hydrocarbon groups. Importantly, the spectrum of Polymer@mPDA contained characteristic peaks from both the polymer and mPDA, confirming the successful loading of the polymer onto the mPDA carrier.

### 2.2 Photothermal Performance Assessment of Polymer@mPDA

To evaluate the PTT properties of mPDA, Polymer, and Polymer@mPDA, we first conducted UV–vis spectroscopy analysis. As shown in Figure 2I, mPDA exhibited low absorption across the entire wavelength range without distinct characteristic peaks. In contrast, Polymer exhibited significant absorption in both the visible (430 nm) and near‐infrared (800–1000 nm) regions. Notably, the absorption profile of Polymer@mPDA largely overlapped with that of Polymer, further confirming the successful combination of Polymer and mPDA and indicating the material's potential for photothermal applications under NIR irradiation. To verify the photothermal performance of Polymer@mPDA, we irradiated aqueous dispersions of mPDA and Polymer@mPDA (100 µg/mL of polymer) with a 980 nm laser. Under irradiation at 1.0 W/cm^2^ for 10 min, the temperature of mPDA reached 38.2°C, while Polymer@mPDA reached 50.5°C (Figure 2J and Figure S3), demonstrating significantly enhanced photothermal efficiency. This property provides a promising basis for the photothermal inactivation of MPXV. We further investigated the photothermal behavior of Polymer@mPDA under different laser power densities and material concentrations. The results revealed notable power‐dependent (Figure 2K) and concentration‐dependent (Figure 2L) temperature increases. Corresponding thermal images (Figures S4 and S5) visually illustrated the temperature changes under these varying conditions. Moreover, Polymer@mPDA maintained stable photothermal performance over five consecutive heating‐cooling cycles under on/off laser switching (Figure 2M and FigureS6), indicating excellent photothermal stability. The photothermal conversion efficiency (*η*) of Polymer@mPDA was calculated based on the heating‐cooling curve and relevant formula, yielding a high value of 57.18% (Figure 2N). Collectively, these results demonstrate that Polymer@mPDA possesses remarkable photothermal efficacy and stability, making it a highly promising photothermal agent for efficient photothermal therapy against MPXV.

### 2.3 Preparation and Characterization of Polymer@mPDA@Gel

**FIGURE 3:**
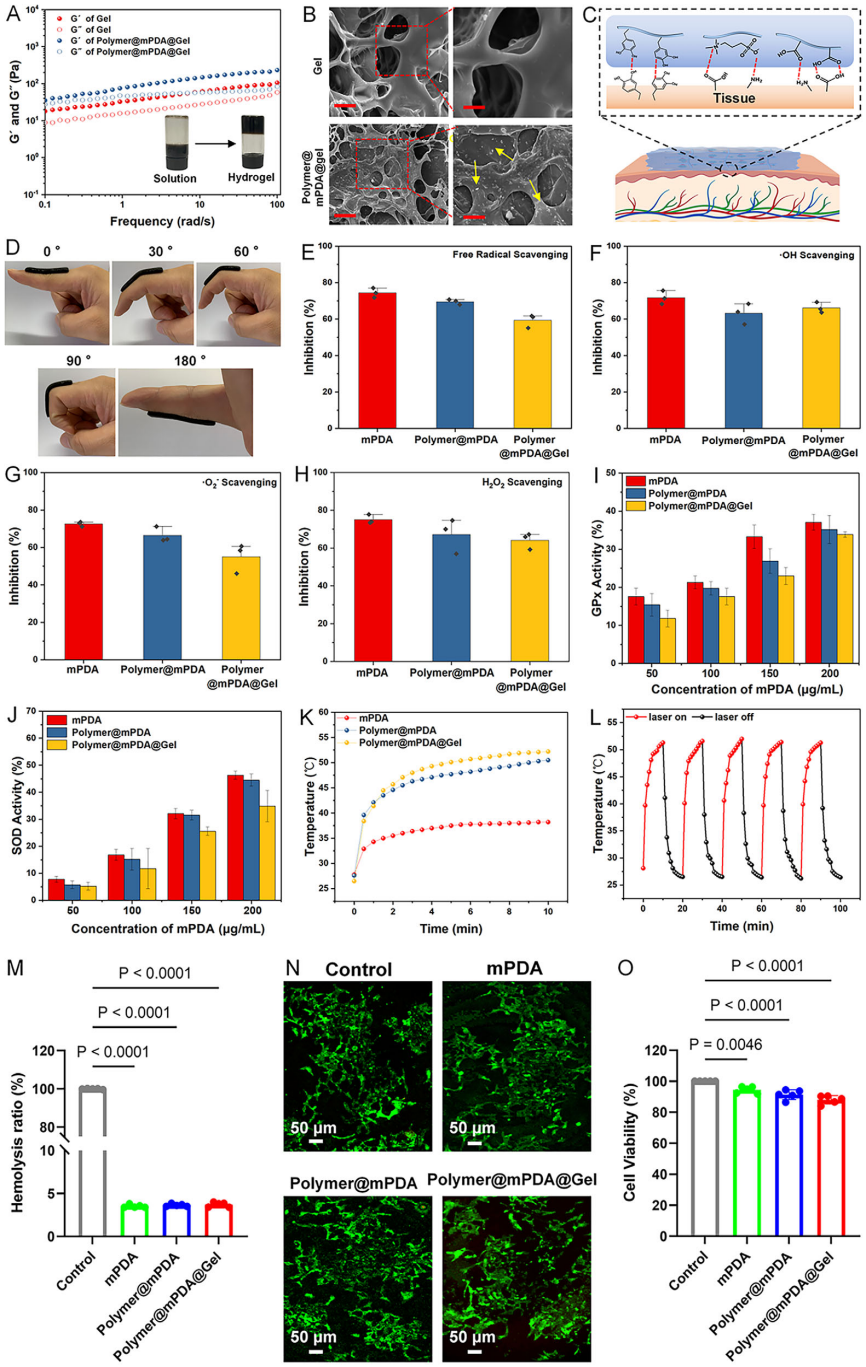
Physicochemical characterization of the hydrogel. (A) G' and G'' of Gel and Polymer@mPDA@Gel under frequency sweep. (B) SEM images of Gel and Polymer@mPDA@Gel. Scale bar, left: 20 µm, right: 10 µm. (C) Adhesion mechanism of Polymer@mPDA@Gel. (D) Photographs demonstrating the skin adhesion effect of Polymer@mPDA@Gel. Scavenging efficiency of mPDA, Polymer@mPDA, and Polymer@mPDA@Gel toward (E) free radicals, (F) ·OH, (G) ·O_2_^−^, and (H) H_2_O_2_. (I) GPx activity and (J) SOD activity of mPDA, Polymer@mPDA, and Polymer@mPDA@Gel at different concentrations. (K) Heating curves of mPDA, Polymer@mPDA, and Polymer@mPDA@Gel under 980 nm laser irradiation (1.0 W/cm^2^). (L) Heating and cooling curves of Polymer@mPDA@Gel over five on‐off laser cycles. (M) Hemolysis rates of mPDA, Polymer@mPDA, and Polymer@mPDA@Gel. (N) Live/dead staining images of Vero E6 cells after 24 h co‐incubation with mPDA, Polymer@mPDA, and Polymer@mPDA@Gel. (O) Statistical analysis of cell viability of Vero E6 cells after 24 h co‐incubation with mPDA, Polymer@mPDA, and Polymer@mPDA@Gel. For E–J, M, and O, data are represented as the mean ± s.d. (*n* ≥ 3). One‐way analysis of variance (ANOVA) with post‐hoc corrections was applied. Experiments in A, B, K, L, and N were repeated independently three times with consistent results, and a representative result is shown for each.

Owing to their protective effects on wounds and unique advantages as drug delivery carriers, hydrogels have been extensively utilized in the development and application of wound dressings. In this study, we developed a functionalized hydrogel (denoted as Polymer@mPDA@Gel) through a hierarchical loading strategy by embedding pre‐synthesized Polymer@mPDA nanoparticles into a p(SBMA‐co‐AA) hydrogel matrix. To investigate the rheological properties of the resulting Polymer@mPDA@Gel, we conducted analyses of its shear‐thinning behavior and viscoelastic moduli. Frequency sweep tests under fixed strain were performed to examine the viscoelastic behavior of both the pure hydrogel (Gel) and the nanocomposite hydrogel (Polymer@mPDA@Gel) (Figure 3A). The storage modulus (G′) reflects the material's resistance to compressive deformation and its mechanical strength. In contrast, the loss modulus (G″) represents the energy dissipated under stress. In both hydrogels, G′ increased slightly with frequency and remained higher than G″, indicating a predominantly elastic, solid‐like behavior and a relatively stable network structure. Moreover, the G′ of Polymer@mPDA@Gel was higher than that of the pure Gel, suggesting that the incorporation of nanoparticles enhanced the elastic network of the hydrogel. This reinforcement is likely attributable to additional interactions, such as hydrogen bonding and physical entanglements, between the nanoparticles and the hydrogel matrix. The shear‐thinning behavior was quantified by fitting the viscosity curves to a power‐law model. As the shear rate (˙γ) increased, the apparent viscosity (*η*) of both hydrogels decreased (Figure S7A), demonstrating typical shear‐thinning behavior alongside an increase in shear stress (Figure S7B). Furthermore, shear stress increased with shear strain, and the nearly overlapping curves of the two hydrogels indicated that the incorporation of Polymer@mPDA nanoparticles had minimal influence on the yield characteristics within the tested strain range (0.1%–100%) (Figure S7C). This suggests that the macroscopic shear deformation resistance of the hydrogel was not significantly altered.

The microstructural morphology of hydrogels is closely related to their drug‐loading efficiency. SEM images revealed that both hydrogels exhibit a porous structure, which provides a favorable environment for the incorporation of Polymer@mPDA. Specifically, the pure Gel displayed a relatively smooth surface, whereas the Polymer@mPDA@Gel showed a rougher texture with distinct particles attached to its surface (Figure 3B). To evaluate the compressive resistance of the hydrogels, axial compression tests were conducted to simulate their mechanical response under pressure (Figure S8A). The stress curves of both hydrogels were largely consistent under strains ranging to 80% (Figure S8B), indicating that the incorporation of Polymer@mPDA did not significantly compromise toughness and that the composite maintained good compressive strength. The Polymer@mPDA@Gel was adhered to porcine skin and subjected to bending and twisting tests. It remained stably attached throughout the mechanical challenges and also demonstrated considerable stretchability (Figure S8C). Adhesion is a crucial property for hydrogels to maintain continuous attachment to wound sites and promote healing. The mPDA component contains abundant catechol groups, which can form hydrogen bonds with polar groups such as hydroxyl and amino groups on skin tissue, in addition to enhancing interfacial interactions via *π*–*π* stacking. Furthermore, the sulfonate groups from SBMA and the carboxyl groups from AA in the hydrogel matrix facilitate electrostatic interactions with tissue surfaces. The synergistic effect of these functional groups contributes to robust tissue adhesion (Figure 3C). A shear adhesion test performed on porcine tissue (Figure S9A) demonstrated that both hydrogels exhibit strong shear adhesive strength (Figure S9B), ensuring secure attachment to the skin while allowing convenient removal and replacement. The Polymer@mPDA@Gel effectively adhered to human finger skin and maintained its adhesion during repeated bending movements, confirming its excellent adhesive performance (Figure 3D). Furthermore, Polymer@mPDA@Gel demonstrates effective adhesion to a variety of biological tissues and synthetic substrates, including ceramic, rubber, metal, glass, and plastic. (Figure S10).

### 2.4 Photothermal and Antioxidant Properties of Polymer@mPDA@Gel

The core pathology of MPXV infection is centered on the skin, where the virus invades keratinocytes and replicates. The inflammatory response accompanying skin injury advances in stages, peaking during the vesicle‐to‐pustule phase with neutrophil aggregation and a surge in inflammatory factors. Uncontrolled inflammation may exacerbate tissue necrosis and delay healing. “Inhibiting viral replication + modulating inflammation” is the key to treatment. Viral suppression reduces cellular damage and blocks the source of injury, laying the groundwork for inflammation control. Modulating inflammation prevents excessive tissue damage while preserving its antiviral functions, thereby creating an environment conducive to skin repair. This synergistic approach breaks the vicious cycle of “viral damage to exacerbated inflammation,” providing dual safeguards for recovery.

Building upon the confirmed excellent photothermal performance of Polymer@mPDA, the photothermal properties of Polymer@mPDA@Gel were further investigated. As shown in Figure 3K, under 980 nm laser irradiation (1.0 W/cm^2^), mPDA exhibited relatively poor photothermal efficiency, while both Polymer@mPDA and Polymer@mPDA@Gel demonstrated significant photothermal effects. Notably, under the same irradiation conditions, Polymer@mPDA@Gel displayed superior photothermal performance compared to Polymer@mPDA. This enhancement is likely due to the hydrogel's three‐dimensional network structure, which facilitates uniform nanoparticle dispersion and prevents aggregation. Additionally, the hydrogel's dense structure effectively reduces heat dissipation, thereby improving photothermal efficiency. This observation was further supported by the heating distribution images (Figure S11). When the three materials were irradiated with the laser, both mPDA and Polymer@mPDA showed broad heat dispersion covering almost the entire area of the EP tube. In contrast, the heating region of Polymer@mPDA@Gel was predominantly concentrated within the laser‐irradiated area. Photothermal stability experiments revealed that Polymer@mPDA@Gel exhibits excellent stability, comparable to that of Polymer@mPDA (Figure 3L and Figure S12).

To evaluate the ROS scavenging ability of Polymer@mPDA@Gel, the total radical scavenging capacity of mPDA, Polymer@mPDA, and Polymer@mPDA@Gel (mPDA concentration of 200 µg/mL) was first tested using Water‐Soluble Tetrazolium (WST‐1). The results showed that over 60% of the radicals were scavenged by Polymer@mPDA@Gel (Figure 3E). Furthermore, tests on three common types of radicals, ·OH, ·O_2_^−^, and H_2_O, revealed that Polymer@mPDA@Gel achieved scavenging rates of 63.7%, 60.6%, and 59.2%, respectively (Figure 3F–H). The activities of glutathione peroxidase (GPx) and superoxide dismutase (SOD) in Polymer@mPDA@Gel were also investigated at varying concentrations. The results demonstrated that both the GPx‐like and SOD‐like activities of Polymer@mPDA@Gel exhibited concentration‐dependent characteristics (Figure 3I,J). Steady‐state kinetic analysis was employed to investigate the enzymatic properties of Polymer@mPDA@Gel in the presence of varying H_2_O_2_ concentrations. The results revealed typical Michaelis‐Menten kinetics (Figure S13). The apparent Michaelis constant (K_m_) and maximum reaction rate (V_max_) of Polymer@mPDA@Gel with H_2_O_2_ as the substrate were determined to be 7.37 mM and 8.52 × 10^−5^ M·s^−1^, respectively, indicating a strong affinity of Polymer@mPDA@Gel for H_2_O_2_.

The catechol moiety within the mPDA framework serves as the core functional unit enabling broad‐spectrum ROS scavenging and triple enzyme‐mimetic activities (SOD and GPx). Leveraging its reversible redox cycling, catechol orchestrates multi‐pathway antioxidation as follows: (1) For SOD‐like activity, reduced catechol sequentially donates single electrons to superoxide anions, facilitating their dismutation into hydrogen peroxide and oxygen, while itself being progressively converted into semiquinone and then o‐benzoquinone. Intracellular GSH can reduce the quinoid structures to regenerate active phenolic hydroxyls, thereby sustaining the catalytic reaction. (2) During GPx‐mimicking processes, o‐benzoquinone reduces hydrogen peroxide and lipid peroxides, being reduced back to catechol. The latter subsequently undergoes dehydrogenation by two GSH molecules to generate GSSG and is reoxidized to the quinone structure. This cyclical process eliminates peroxides and halts the lipid peroxidation chain reaction; concurrently, the phenolic hydroxyls can directly scavenge ·OH, achieving non‐catalytic radical quenching.

The composite hydrogel matrix modulates the antioxidant performance of mPDA across multiple dimensions: its three‐dimensional crosslinked network suppresses nanoparticle aggregation, ensuring full exposure of catechol active sites; its porous structure entraps localized ROS and enriches endogenous GSH, prolonging substrate–active site contact time and guaranteeing the continuity of the catalytic cycle; and its polymer chains encapsulate the particles via intermolecular interactions, preventing irreversible oxidative deactivation of catechol, while hydrophilic buffering groups stabilize the pH of the inflammatory microenvironment to maintain optimal conditions for enzyme‐mimetic catalysis. In summary, the hydrogel provides carrier effects encompassing stable dispersion, microenvironmental buffering, and long‐term protection, thereby amplifying the catechol‐mediated triple enzyme‐mimetic pathways of mPDA. The synergy between the two constructs a highly efficient and long‐lasting antioxidant composite system.

### 2.5 Validation of Antiviral and Anti‐Inflammatory Activities in Vitro

Given that acceptable biocompatibility is a prerequisite for advancing the application of biomaterials, we first evaluated the blood compatibility of mPDA, Polymer@mPDA, and Polymer@mPDA@Gel; the hemolysis rates in all material groups were below 5%, demonstrating that mPDA, Polymer@mPDA, and Polymer@mPDA@Gel possess favorable hemocompatibility (Figure 3M and Figure S14). Meanwhile, we investigated the cytocompatibility of Polymer@mPDA@Gel by measuring the viability of Vero E6 cells after incubation with leachates of mPDA, Polymer@mPDA, and Polymer@mPDA@Gel. As shown in Figure 3N, Vero E6 cells maintained normal growth after 24 h of incubation with the leachates. Furthermore, CCK‐8 results (Figure 3O) indicated that the cells retained high viability following co‐incubation with the materials. Furthermore, to verify the long‐term biosafety of polymer@mPDA@gel in mice, the composite was administered intraperitoneally. After 21 days, the major organs (heart, liver, spleen, lungs, and kidneys) were collected for pathological examination. Histopathological analysis revealed no significant toxic effects (Figure S15).

**FIGURE 4:**
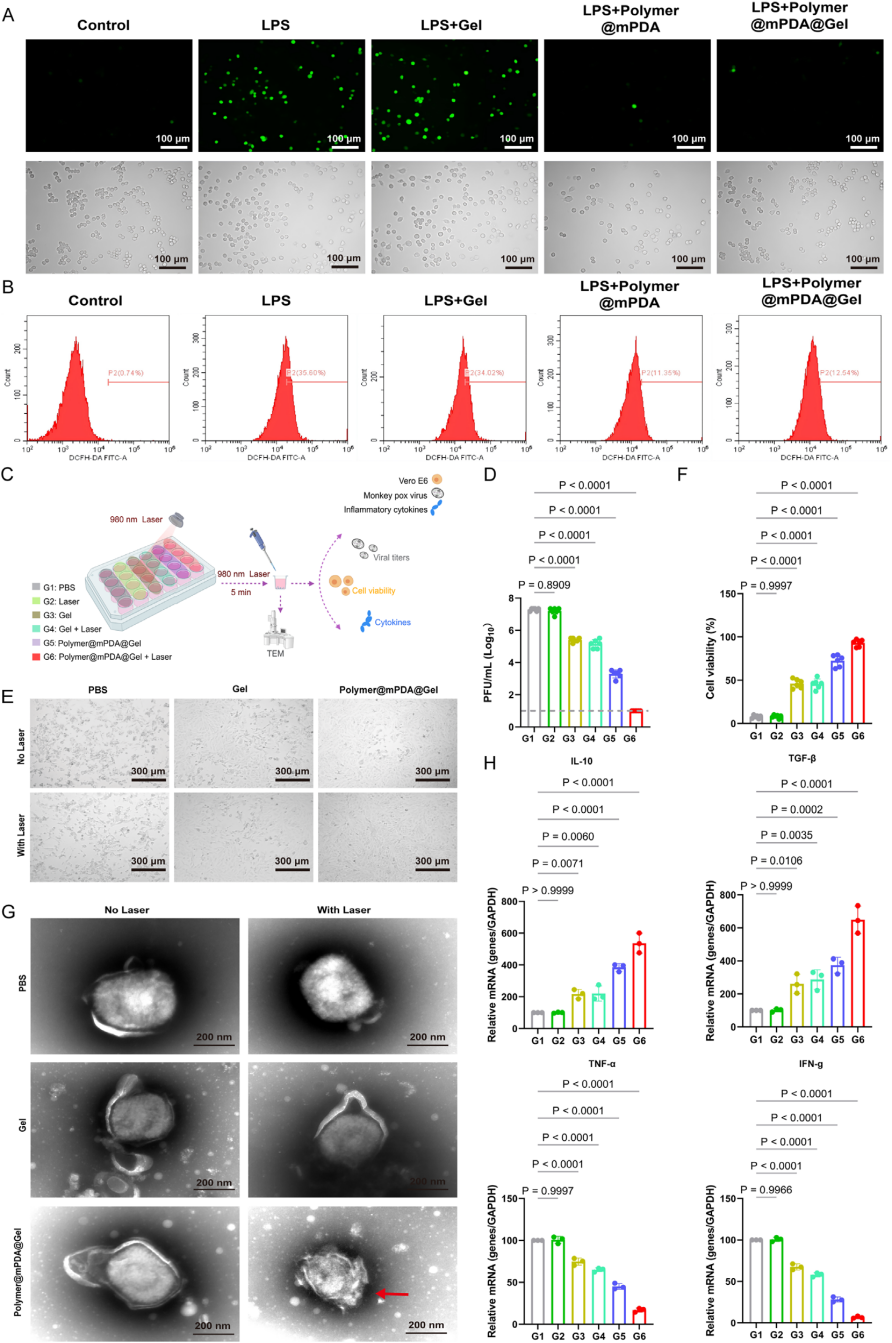
Antiviral and anti‐inflammatory efficacy of “Polymer@mPDA@Gel” in vitro. (A) Fluorescence images of macrophages from different groups after LPS stimulation, followed by DCFH‐DA staining. (B) Flow cytometry analysis of macrophages from different groups after LPS stimulation, followed by DCFH‐DA staining. (C) Schematic of in vitro experimental design. A total of 200 µL DMEM containing 10^6^ plaque‐forming units (PFU) of MPXV was co‐incubated with PBS (G1), PBS + laser (G2), Gel (G3), Gel + laser (G4), Polymer@mPDA@Gel (G5), or Polymer@mPDA@Gel+laser (G6) for 30 min with or without subsequent 980 nm laser (1.0 W/cm^2^) illumination. (D) Viral titers determined by plaque assay following respective treatments. (E) Micrographs of Vero E6 cells 24 h post‐infection with treated viruses. (F) Viability of Vero E6 cells infected with treated viruses, assessed by CCK‐8 assay. (G) TEM images of MPXV particles. Red arrowheads highlight structural disintegration of viruses. (H) Relative mRNA expression of anti‐inflammatory (IL‐10 and TGF‐β) and pro‐inflammatory (TNF‐α and IFN‐g) cytokines in macrophages exposed to differential pre‐treatment of the MPXV. For D, F, and H, data are represented as the mean ± s.d. (*n* ≥ 3). One‐way analysis of variance (ANOVA) with post‐hoc corrections was applied. Experiments in A, B, E, and G were repeated independently three times with consistent results, and a representative result is shown for each.

The intracellular ROS‐scavenging property of mPDA was assessed using. As shown in Figure 4A, after staining with DCFH‐DA, strong fluorescence was observed in lipopolysaccharide (LPS)‐stimulated macrophages, indicating substantial ROS production. In contrast, fluorescence intensity was markedly reduced in groups treated with mPDA, indicating effective intracellular ROS clearance. This finding was further corroborated by flow cytometry analysis (Figure 4B). Following LPS stimulation, the proportions of macrophages with high ROS expression were 35.60% (LPS group) and 34.02% (LPS + Gel group). In contrast, those in the mPDA‐containing groups were significantly lower, at 11.35% (LPS + Polymer@mPDA) and 12.54% (LPS + Polymer@mPDA@Gel). Regarding the observation that the ROS‐scavenging efficiency of Polymer@mPDA@Gel was lower than that of Polymer@mPDA, the possible reason is that free Polymer@mPDA nanoparticles can diffuse freely, rapidly penetrate the cell membrane, and enter macrophages, where the abundant catechol groups efficiently scavenge intracellular ROS. In contrast, when Polymer@mPDA is encapsulated within the three‐dimensional network of the p(SBMA‐co‐AA) hydrogel, the polymer chains form a physical diffusion barrier that slows down the penetration of nanoparticles into intercellular spaces and cells. Consequently, at the same incubation time, fewer functional particles enter the cells, resulting in a lower total number of intracellular antioxidant active sites and thus more residual ROS, with a slight increase in the proportion of ROS‐positive cells.

To evaluate the antiviral activity of Polymer@mPDA@Gel under laser irradiation, MPXV was co‐incubated with PBS, Gel, or Polymer@mPDA@Gel for 30 min, followed by exposure to a 980 nm laser (1.0 W/cm^2^) in designated groups (Figure 4C). As shown in Figure 4D, all groups, except the PBS group, exhibited varying levels of viral clearance. Notably, the combination of Polymer@mPDA@Gel and laser irradiation resulted in near‐complete viral inactivation, an effect we attribute to the photothermal activity of the incorporated cationic conjugated polymer (Polymer@mPDA). Host cells (Vero E6) were then infected with the virus and subjected to the different pretreatment conditions. After 24 h, significant cytopathic effects and reduced cell viability were observed in both the “no laser” and “PBS + laser” groups. In contrast, cells exposed to “Gel ± laser” showed partial protection, whereas the “Polymer@mPDA@Gel + laser” group completely prevented host cell death (Figure 4E,F). TEM further revealed distinct morphological changes: intact MPXV particles exhibited a characteristic brick‐like shape with dense internal protein packing, whereas laser‐treated Polymer@mPDA@Gel induced structural disassembly, resulting in loosely packed viral proteins and disrupted virions (Figure 4G). Under 980 nm laser irradiation, Polymer@mPDA@Gel generates localized hyperthermia, which first disrupts the intermolecular forces of the outer phospholipid envelope of the virus, causing membrane damage and thermal denaturation of envelope glycoproteins, thereby abolishing its host cell infectivity. Continuous temperature elevation further breaks the hydrogen bonds, hydrophobic interactions, and disulfide bonds of the capsid proteins, inducing irreversible unfolding and loosening of the capsid protein arrangement, leading to disassembly of the overall viral particle structure. The heat simultaneously disrupts the binding between DNA and nucleoproteins, breaks the hydrogen bonds of nucleic acid bases, and causes fragmentation of the genomic double strands, resulting in complete inactivation of the viral genetic material and inability to complete replication and proliferation [27, 49]. Additionally, when macrophages were exposed to virus pretreated with “Polymer@mPDA@Gel + laser”, no upregulation of pro‐inflammatory cytokines (IFN‐g and TNF‐α) was detected. Instead, we observed enhanced expression of anti‐inflammatory mediators (TGF‐β and IL‐10) (Figure 4H). This dual capacity for viral clearance and inflammation control highlights the system's multifunctional utility in vitro, supporting progression to in vivo evaluation of Polymer@mPDA@Gel + laser.

### 2.6 Antiviral Activity, Wound‐Healing Promotion, and Transmission Blockade in a Murine Tail Infection Model

**FIGURE 5:**
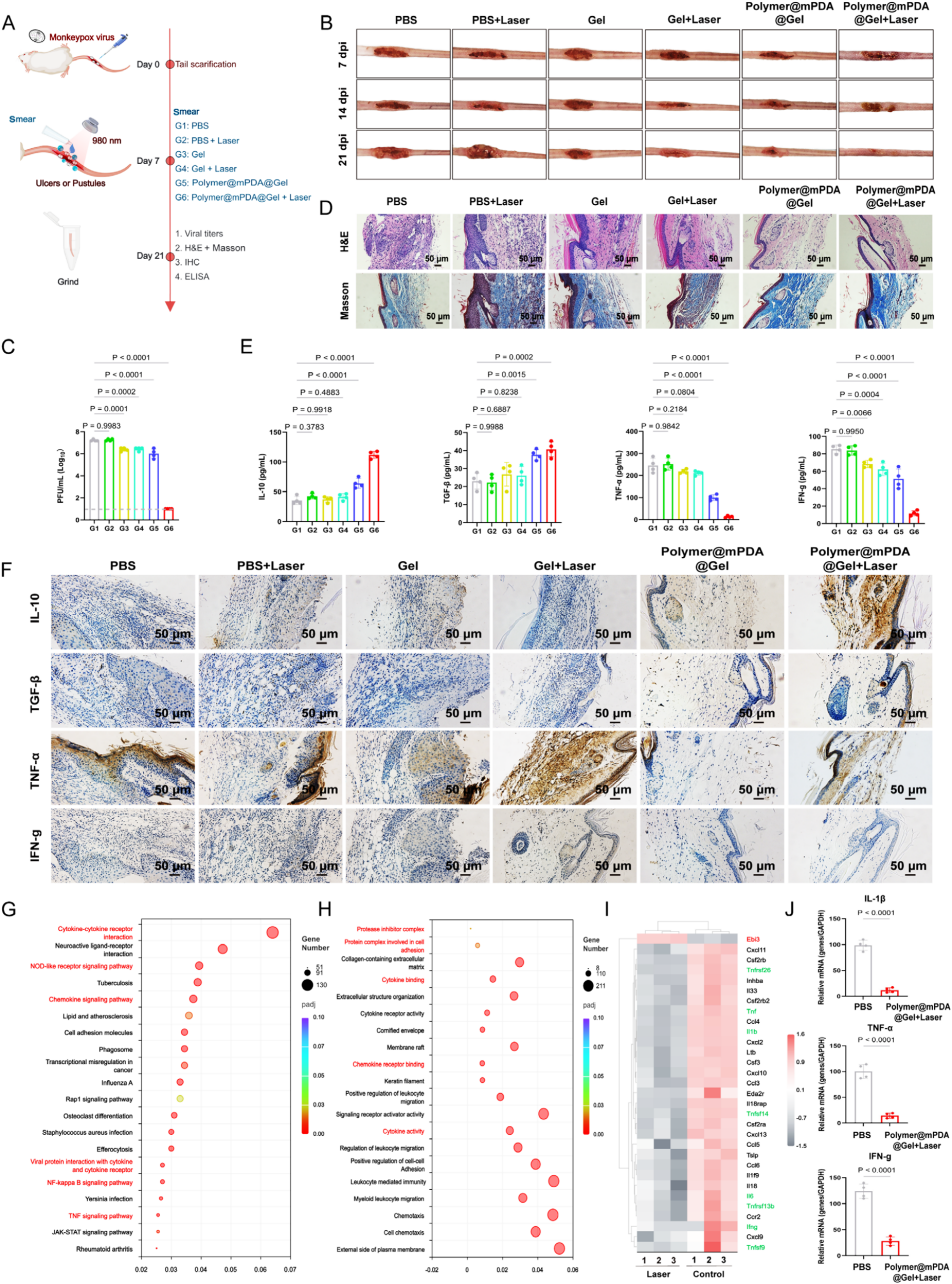
Antiviral activity and wound healing promotion efficacies of “Polymer@mPDA@Gel” in a murine tail infection model. (A) Schematic of the murine tail scarification model. Six‐week‐old mice were infected with 20 µL DMEM containing 10^4.5^ PFU of MPXV via scarification. On 7 dpi, mice with visible scabs were randomly assigned to the following treatment groups: PBS, Gel, or Polymer@mPDA@Gel, each with or without 980 nm laser irradiation. Treatments were administered, and mice were monitored for 14 days. (B) Representative images of tail scabs before and after treatment. (C) Viral loads in tail tissue homogenates were determined by PFU assay. (D) H&E and Masson staining of tail lesion tissues. (E‐F) Cytokine expression profiles (IL‐10, TGF‐β, TNF‐α, IFN‐g) in tail tissues assessed by IHC staining and ELISA. (G–I) Transcriptomic analysis of skin tissues (*n* = 3) from PBS‐ and Polymer@mPDA@Gel + laser‐treated groups at 6 dpi: KEGG pathway analysis (G), Gene Ontology (GO) enrichment (H), and heatmap (I) of differentially expressed immune‐related genes (fold change ≥ 2, *p* ≤ 0.05). (J) Relative mRNA expression levels of IL‐1β, IFN‐α, and TNF‐α in tail tissues. Data in C, E, and J are presented as mean ± s.d. (*n* = 4). Significance was determined using one‐way ANOVA with post hoc testing. Experiments in B, D, and F were repeated three times independently with consistent results; representative data are shown.

MPXV infection typically manifests as localized, ulcerative, or pustular lesions on the skin and mucous membranes. Tail scarification has been established as a physiologically relevant model for orthopoxvirus infection, as evidenced in prior smallpox vaccine studies [50]. Here, we employed a murine tail scarification model infected with MPXV to recapitulate the pustular pathology characteristic of human monkeypox (Figure 5A). By 7 dpi, SCID mice developed pronounced tail scabbing, indicative of sustained viral replication and robust local inflammatory responses (Figure 5B). We systematically evaluated the antiviral, anti‐inflammatory, and wound‐healing properties of Gel and Polymer@mPDA@Gel under laser irradiation. By 21 dpi, mice treated with Polymer@mPDA@Gel plus laser exhibited nearly complete wound resolution (Figure 5B), whereas other groups maintained persistent ulceration, scabbing, and tissue necrosis. Correspondingly, viral loads in tail tissues from the Polymer@mPDA@Gel + laser group fell below the detection threshold, whereas all other groups showed no significant viral clearance (Figure 5C).

Histopathological assessment further supported these findings. H&E staining revealed epidermal regeneration and reduced inflammatory cell infiltration, while Masson's trichrome staining demonstrated enhanced collagen deposition, consistent with structurally and functionally restored skin in “Polymer@mPDA@Gel + laser” (Figure 5D). Immunological profiling via ELISA and immunohistochemistry (IHC) indicated that Polymer@mPDA@Gel + laser treatment induced a distinct anti‐inflammatory response, upregulating IL‑10 and TGF‑β by approximately 3‑fold and 2‑fold, respectively, and reducing TNF‑α and IFN‑g levels by over 80% (Figure 5E,F). Transcriptomic analysis of lesion tissues provided mechanistic insights: KEGG pathway enrichment indicated the suppression of pro‐inflammatory cascades, including TNF signaling and cytokine‐cytokine receptor interactions (Figure 5G). A differential gene expression heatmap further confirmed downregulation of inflammatory mediators (e.g., IL‑6, IL‑1β, and IFN‑g) and upregulation of the anti‑inflammatory gene Ebi3 (Figure 5I), findings corroborated by Gene Ontology (GO) analysis (Figure 5H). As shown in Figure 5J, qRT‐PCR results were consistent with RNA‐seq findings.

**FIGURE 6:**
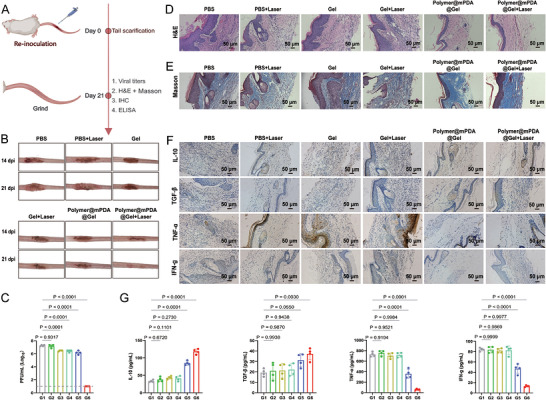
Transmission Blockade Efficacy in a Murine Reinoculation Model. (A) Schematic diagram of the mouse tail reinoculation model. (B) Representative images of tail lesions in reinoculated SCID mice at 14 and 21 dpi. (C) Viral titers in tail tissue homogenates measured by PFU assay. (D,E) Histological evaluation of tail lesions via H&E and Masson's trichrome staining. (F,G) Cytokine expression profiles (IL‐10, TGF‐β, TNF‐α, and IFN‐g) in tail tissues were assessed by IHC staining and ELISA. Data in C and G are presented as mean ± s.d. (*n* = 4). Significance was determined using one‐way ANOVA with post hoc testing. Experiments in B, D, E, and F were repeated three times independently with consistent results; representative data are shown.

Interrupting viral transmission is a cornerstone of MPXV outbreak containment. Motivated by preliminary observations in our murine model (Figure 5), we conducted a dedicated reinoculation study to assess the transmission‐blocking capacity of the Polymer@mPDA@Gel‐based treatment rigorously. Tail lesion homogenates from each treatment group were reinoculated into healthy SCID mice via scarification (Figure 6A). Homogenates from the Polymer@mPDA@Gel + laser group induced no visible lesions in recipient animals (Figure 6B). Viral titers in these mice remained below the detection limit (Figure 6C). In contrast, homogenates from all other groups provoked severe ulceration and high viral loads (>10^6^ PFU/mL). Histopathological evaluation corroborated these results: only tissues from the Polymer@mPDA@Gel + laser group maintained normal architecture and collagen deposition, with no signs of necrosis or inflammatory infiltration, whereas other groups exhibited substantial dermal disruption (Figure 6D,E). Immunological profiling (IHC and ELISA) further underscored the absence of pro‐inflammatory activation in mice receiving Polymer@mPDA@Gel + laser‐treated homogenates, with minimal TNF‐α and IFN‐g expression and elevated IL‐10 and TGF‐β levels (Figure 6E,F). In conclusion, our findings demonstrate that the Polymer@mPDA@gel + laser treatment not only eliminates MPXV from infected tissues and mitigates inflammation but also fully abolishes viral infectivity.

### 2.7 Antiviral Activity, Wound‐healing Promotion, and Transmission Blockade in a Rabbit Skin Model

**FIGURE 7:**
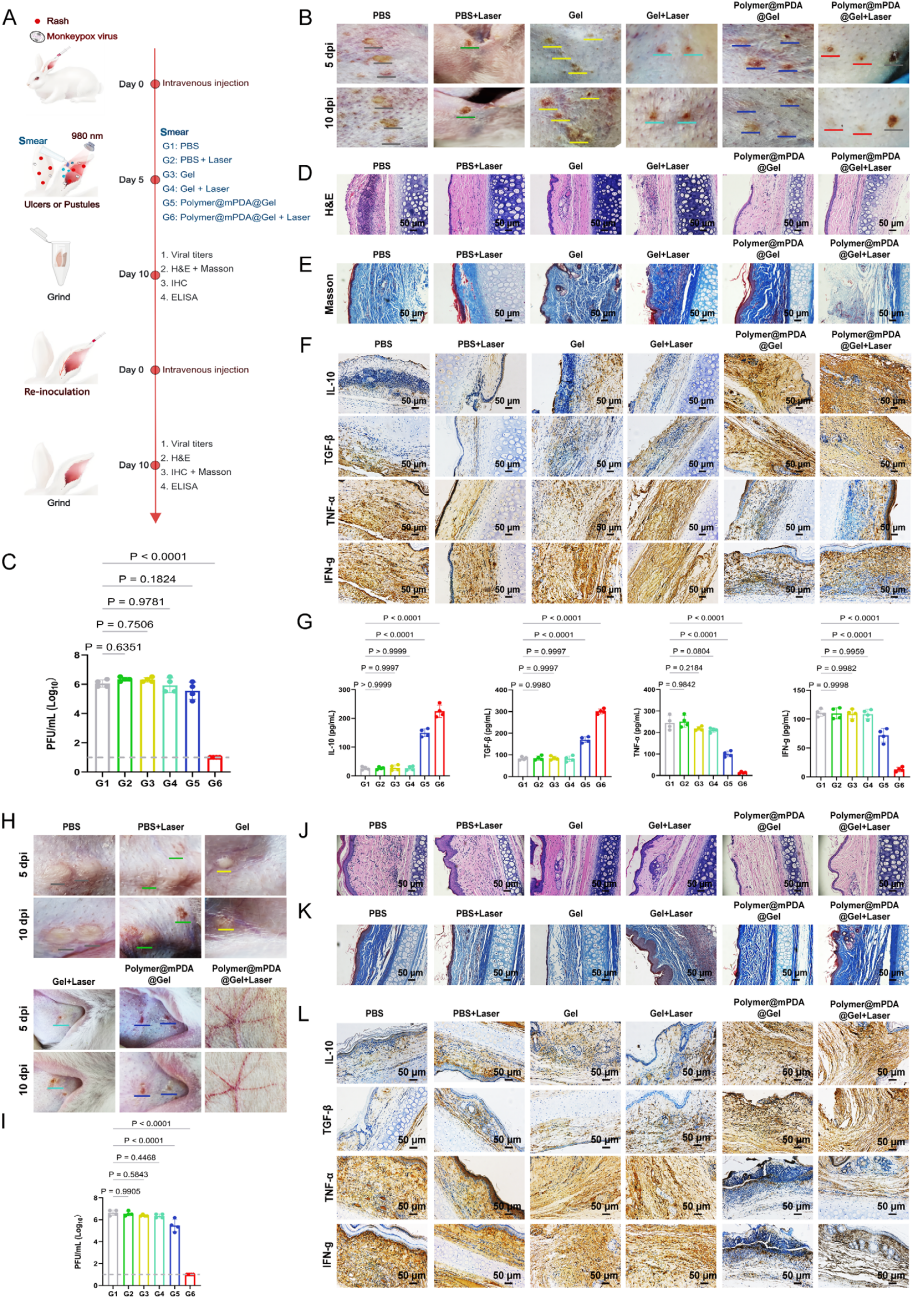
Antiviral activity, wound healing promotion, and transmission blockade efficacies of “Polymer@mPDA@Gel + laser” in a rabbit skin model. (A) Schematic of the in vivo rabbit skin model. Adult New Zealand white rabbits infected with 10^6^ PFU of MPXV were treated at 5 dpi with PBS, Gel, or Polymer@mPDA@Gel, each with or without 980 nm laser irradiation. Treatments were administered, and rabbits were monitored for 5 days. Tissue homogenates from treated rabbits were subsequently reinoculated into healthy rabbits and monitored for 10 days. (B) Representative images of the ear before and after treatment. (C) Viral loads in ear tissue homogenates measured by PFU assay. (D,E) H&E and Masson staining of ear tissues. (F,G) Cytokine expression profiles (IL‐10, TGF‐β, TNF‐α, IFN‐g) in ear tissues assessed by IHC staining and ELISA. (H) Representative images of ears from reinoculated rabbits at 5 and 10 dpi. (I) Viral loads in ear homogenates after reinoculation. (J,K) Histological evaluation of ear lesions via H&E and Masson's trichrome staining of reinoculated rabbits. (L) Cytokine (IL‐10, TGF‐β, TNF‐α, and IFN‐g) levels in reinoculated rabbits via IHC and ELISA. Data in C, G, and I are presented as mean ± s.d. (*n* = 4). One‐way ANOVA was used to determine significance, with post‐hoc testing. Experiments in B, D, E, F, H, J, K, and L were repeated three times independently with consistent results; representative data are shown.

The rabbit model was selected for this study due to its physiological relevance to humans in skin irritation responses. To evaluate the therapeutic efficacy of our nanocomposite, we established a systemic MPXV infection by intravenous inoculation via the ear vein, building upon recent models that recapitulate key clinical features of monkeypox, including rash, fever, and genital lesions (Figure 7A). At 5 dpi, following the onset of dermal rash, animals received topical administration of PBS, Gel, or Polymer@mPDA@Gel, with or without 980 nm laser irradiation. Within five days of treatment, only rabbits treated with Polymer@mPDA@Gel plus laser exhibited complete rash resolution and full wound re‐epithelialization (Figure 7B). Viral titers in rash tissues were nearly undetectable in this group, confirming efficient viral clearance (Figure 7C). Histological assessment further demonstrated that the combination therapy promoted epidermal regeneration and organized collagen deposition, in contrast to the necrotic and disorganized tissue architecture seen in control groups (Figure 7D,E). Immune profiling corroborated earlier murine data, revealing an approximately 10‐fold increase in IL‐10 and a 3‐fold upregulation of TGF‐β, accompanied by a greater than 90% reduction in TNF‐α and IFN‐g (Figure 7F,G).

Reinoculation studies provided further evidence of the nanocomposite's antiviral efficacy (Figure 7H–L). Homogenates from all other treatment groups remained infectious, inducing lesions (Figure 7H), sustaining high viral loads (Figure 7I), and disrupting tissue architecture in healthy rabbits (Figure 7J,K). In contrast, those from the Polymer@mPDA@Gel + laser group were non‐infectious and elicited a modulated immune response upon challenge (Figure 7L and Figure S16), confirming a consistent inter‐species transmission blockade. Together, these findings support the translational promise of Polymer@mPDA@Gel‐mediated photothermal therapy as an integrated strategy for treating and preventing mpox transmission.

### 2.8 Antiviral Activity and Wound‐healing Promotion in a Primate Skin Model

**FIGURE 8:**
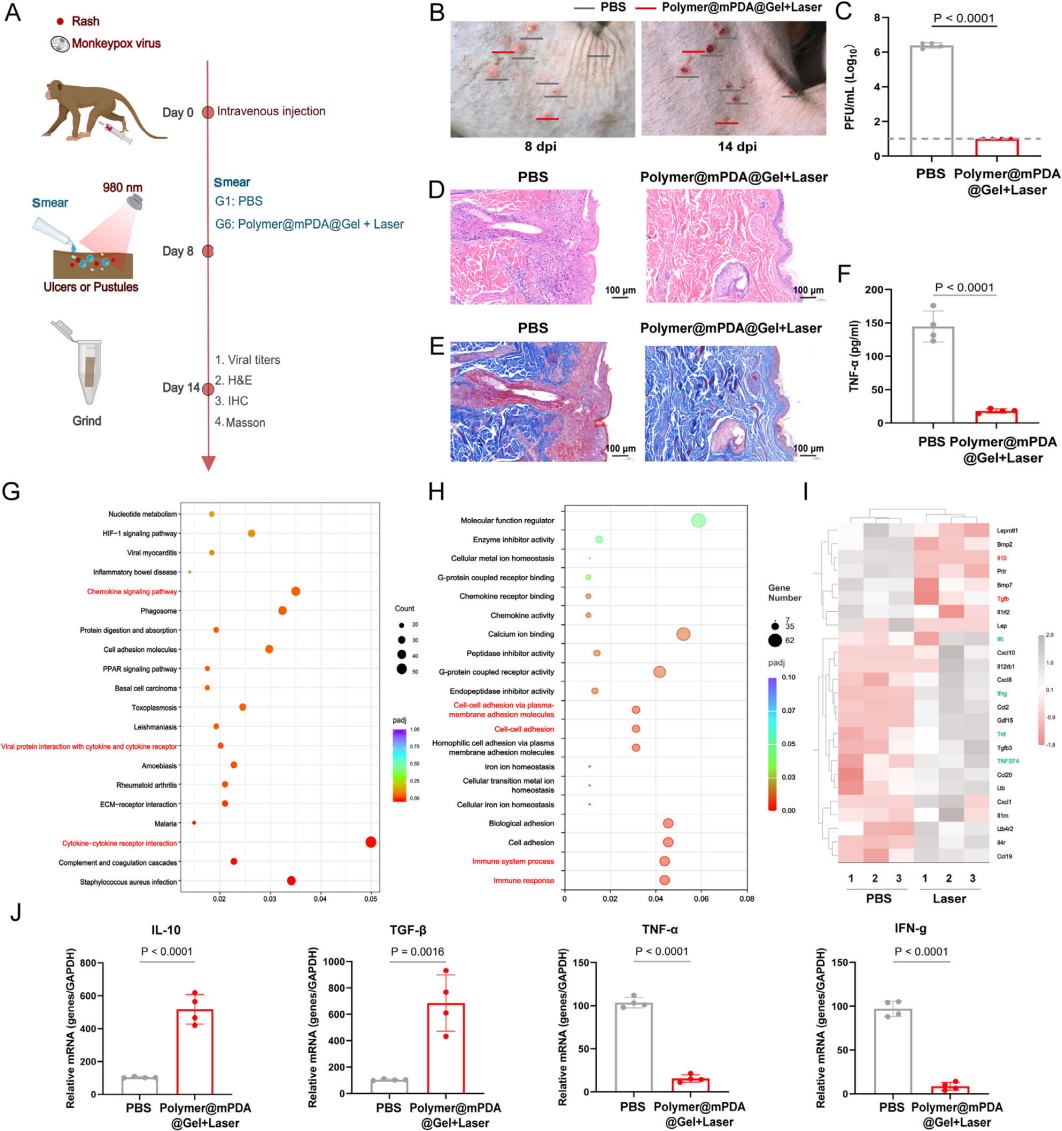
Evaluation of Polymer@mPDA@Gel for antiviral and wound‐healing efficacy in a primate model of MPXV infection. (A) Experimental timeline: Cynomolgus macaques (*n* = 6) were intravenously inoculated with MPXV (10^6^ PFU). At 8 dpi, animals with skin ulcers/pustules were randomized to receive topical PBS or Polymer@mPDA@Gel, followed by 980 nm laser irradiation and 6‐day monitoring. (B) Representative clinical photographs of skin lesions pre‐ and post‐treatment. (C) Viral titers in skin tissues measured by PFU assay. (D‐E) Histological analysis of skin lesions by H&E staining and Masson's trichrome staining. (F) TNF‐α levels in skin tissue homogenates quantified by ELISA. (G) KEGG pathway enrichment analysis of differentially expressed genes (DEGs). (H) GO functional enrichment analysis of DEGs. (I) Heatmap of DEGs associated with immune regulation. (J) Relative mRNA expression levels in skin lesions. Data in C, F, and J are presented as mean ± s.d. (*n* = 4). One‐way ANOVA was used to determine significance, with post‐hoc testing. Experiments in B, D, and E were repeated three times independently with consistent results; representative data are shown.

The cynomolgus macaque model recapitulates key clinical manifestations of human MPXV infection, including characteristic cutaneous and mucosal lesions [51]. To evaluate the translational potential of our nanocomposite hydrogel, we intravenously inoculated six cynomolgus macaques with MPXV (Figure 8A). At 8 dpi, macaques with generalized rash were randomized into two groups that received either topical PBS or Polymer@mPDA@Gel with 980 nm laser irradiation. Within six days post‐treatment, animals in the Polymer@mPDA@Gel + laser group showed complete pustule resolution and accelerated wound re‐epithelialization. In contrast, PBS‐treated lesions progressed with severe tissue damage (Figure 8B). PFU assays demonstrated near‐complete viral clearance in the treatment group, in contrast to sustained high viral loads in controls (Figure 8C). Histological evaluation (H&E, Masson, and IHC staining) confirmed that the combination therapy significantly suppressed diffuse inflammation and tissue necrosis compared to the PBS‐treated groups (Figure 8D,E). Systemic analysis further supported these findings: ELISA revealed a 90% reduction in TNF‐α (Figure 8F), indicating potent anti‐inflammatory activity. Transcriptomic profiling (Figure 8G–8I) identified downregulation of pro‐inflammatory genes (e.g., IL‐6, TNF‐α, and IFN‐g) and concomitant upregulation of anti‐inflammatory and repair‐associated genes (IL‐10 and TGF‐β) in the combination therapy group. As shown in Figure 8J, qRT‐PCR results were consistent with RNA‐seq findings. Together, these results establish the Polymer@mPDA@Gel photothermal therapy as a promising candidate against human monkeypox, demonstrating integrated antiviral, anti‐inflammatory, and tissue‐repairing functions that address key aspects of the disease.

Clinically, mainstream anti‐MPXV small‐molecule drugs such as tecovirimat exert antiviral effects by interfering with viral replication‐related proteins [52]. However, these chemical agents require multiple rounds of long‐term oral or local administration over 7–14 days to reach effective viral clearance, and long‐term single‐target chemical inhibition inevitably raises the risk of MPXV mutation and subsequent drug resistance [53]. Moreover, pure antiviral drugs lack biological activity to promote damaged skin repair; MPXV‐induced skin ulcers often suffer from persistent inflammation and delayed re‐epithelialization after viral suppression, leading to prolonged recovery periods and scar formation [54]. In contrast, most previously reported photothermal antiviral materials rely on single photothermal ablation to inactivate viruses, which achieves satisfactory instant viral killing capacity yet lacks wound microenvironment regulation functions. Such materials cannot continuously modulate local inflammation or supply bioactive components for skin regeneration, resulting in separated antiviral and wound repair processes and limited comprehensive therapeutic performance [55]. Benefiting from the integration of biocompatible hydrogel carriers and photothermal functional components, our engineered system exhibits prominent comprehensive advantages. First, localized mild photothermal stimulation achieves efficient MPXV inactivation within a single short‐duration irradiation session, significantly shortening the overall treatment time compared with clinical chemical drugs. Second, photothermal therapy eliminates viruses via irreversible physical damage to the viral envelope and internal genetic materials, rather than acting on specific viral protein targets, which fundamentally eliminates the selective pressure driving MPXV drug resistance mutations. Third, the hydrophilic hydrogel scaffold maintains a moist wound microenvironment, releases anti‐inflammatory active substances, and facilitates keratinocyte migration and collagen deposition, enabling simultaneous viral elimination and accelerated full‐thickness skin wound healing without secondary scarring. Overall, this synergistic photothermal antiviral hydrogel breaks the functional limitations of single clinical antiviral drugs and conventional photothermal materials, offering a safe, rapid, drug‐resistance‐free combinatorial therapeutic strategy for MPXV skin lesions.

## 3 Conclusion

In this study, a multifunctional antiviral hydrogel (Polymer@mPDA@Gel) was prepared via one‐step co‐assembly, integrating cationic conjugated polymers, mesoporous polydopamine, and an adhesive gel matrix. This hydrogel can closely conform to the irregular skin and mucosal lesions of monkeypox. Local photothermal therapy neither induces systemic toxicity nor causes side effects, thereby circumventing the drug resistance and toxicity issues associated with existing oral antiviral drugs. The material simultaneously achieves integrated intervention effects, including virus inactivation, anti‐inflammatory action, and promotion of tissue repair. Its efficacy in eliminating viruses within lesions and blocking contact transmission has been verified in multiple animal models, including mice, rabbits, and cynomolgus monkeys, and its material components exhibit good biocompatibility. However, this technology still has several limitations in clinical application: making it difficult to fully cover deep or concealed lesions, and it can only locally eliminate viruses in skin lesions, without addressing systemic viremia. Therefore, future research should combine this approach with systemic antiviral drugs for synergistic therapy and advance to human clinical trials to determine the optimal drug administration and treatment regimens.

## 4 Experimental Section

### 4.1 Preparation of the PDA and mPDA NPs

The nonporous PDA NPs were prepared via oxidative self‐polymerization of dopamine in an alkaline solution. First, 0.6 mL of ammonia, 8 mL of ethanol, and 18 mL of deionized water were mixed and stirred until a uniform solution was formed. Then, 0.1 g of dopamine hydrochloride was added to the above solution and stirred at 1000 rpm for 9 h at 70°C. At the end of the reaction, 60 mL of acetone was added for precipitation, which was then allowed to stand for 10 min. The NPs were harvested by centrifugation and dried for use.

The mPDA NPs were prepared using the soft template method. First, 0.36 g of F127 and 0.36 g of TMB were dissolved in a mixed solution of 65 mL of deionized water and 60 mL of ethanol by stirring at room temperature for 30 min. Then, 90 mg of TRIS was dissolved in 10 mL of deionized water, which was added to the above solution and stirred for 10 min. Next, 60 mg of dopamine hydrochloride was added and stirred at 1000 rpm for 24 h. After the reaction was completed, the supernatant was removed by centrifugation at 15 000 rpm for 15 min, and the obtained precipitate was washed with a mixture of ethanol and acetone solution (v/v, 2:1) three times to remove the template and then dried for use.

### 4.2 Loading of Polymer Into the mPDA NPs

The polymer was loaded into mPDA using a physical adsorption method. Briefly, 1 mg/mL polymer DMSO solution was slowly added dropwise to a suspension of mPDA and stirred overnight at room temperature. After stirring, the mixture was centrifuged at 10 000 rpm for 15 min, and the precipitate was collected to obtain the polymer‐loaded mPDA.

### 4.3 Preparation of the Polymer@mPDA@Gel

Polymer@mPDA@Gel was prepared using a simple one‐pot synthesis method. Briefly, 0.6 g of AA (12 wt.%) and 0.5 g of SBMA (10 wt.%) were sequentially added to 5 mL of deionized water, followed by magnetic stirring until both monomers were completely dissolved. Then, Polymer@mPDA was added to the mixed solution and stirred to ensure uniform dispersion. Subsequently, MBAA (0.4 wt.%), APS (0.2 wt.%), and TEMED (5 µL) were added to the mixture, and stirring continued until complete dissolution. The resulting solution was degassed under vacuum and immediately transferred into a mold, followed by in‐situ polymerization at 60°C for 3 h to form Polymer@mPDA@Gel.

### 4.4 Cell Culture

Vero E6 and RAW 264.7 cells were maintained in Dulbecco's Modified Eagle Medium (DMEM) supplemented with 10% fetal bovine serum (FBS), 80 U/mL penicillin, and 80 µg/mL streptomycin. Cells were incubated at 37 °C in a humidified atmosphere containing 5% CO_2_.

### 4.5 Cytotoxicity Assessment by CCK‐8 Assay

The cytotoxicity of Gel and Polymer@mPDA@Gel toward Vero E6 cells was evaluated using the CCK‐8 assay. Briefly, cells were seeded in 96‐well plates at a density of 1 × 10^4^ cells per well and allowed to adhere overnight at 37 °C. The culture medium was then replaced with PBS leaching solutions of Gel or Polymer@mPDA@Gel. After 24 h of incubation, 10 µL of CCK‐8 solution (Nanjing Keygen Biotech Co., Ltd) was added to each well, and cells were incubated for an additional 4 h. Absorbance was measured at 450 nm using a multi‐mode microplate reader.

### 4.6 In Vitro Cytotoxicity

A calcein‐AM/propidium iodide (PI) double‐staining assay was conducted to assess cell viability further. Vero E6 cells were treated with PBS leaching solutions of Gel or Polymer@mPDA@Gel for 24 h. After two washes with PBS, cells were incubated with a staining solution containing 2 µM Calcein‐AM and PI (Nanjing Keygen Biotech Co., Ltd) for 30 min. Following another PBS wash, live and dead cells were visualized using fluorescence microscopy.

### 4.7 Virus and Animal Models

The Monkeypox virus strain (GenBank: PP778666.1), isolated from a patient in Guangzhou, was propagated and titrated in Vero E6 cells. All MPXV‐related experiments were conducted under Biosafety Level 3 (BSL‐3) conditions. Animal procedures were approved by the Scientific Ethics Committee of Chinese Laboratory Animals and the Animal Welfare and Ethics Committee of the Institute of Veterinary Medicine, Changchun Veterinary Research Institute (IACUC permit no. AMMS‐1‐2023‐041).

### 4.8 Statistical Analysis

All quantitative data are presented as mean ± standard deviation (s.d.) from at least three independent experiments (*n*≥3). Differences between the two groups were assessed using two‐tailed unpaired Student's t‐tests, while multiple comparisons were analyzed by one‐way ANOVA with post‐hoc correction. A *p*‐value < 0.05 was considered statistically significant. All analyses were performed using GraphPad Prism 5.

## Author Contributions

**Xiaolong Zhu**: methodology, Writing – original draft, formal analysis, data curation. **Jiaxin Tian**: investigation, data curation, writing – original draft, methodology. **Ning Shi**: writing – original draft, writing – review and editing, conceptualization, data curation, project administration, supervision. **Yuanyuan Li**: supervision, conceptualization, writing – review and editing, project administration. **Xiao Li**: investigation, resources, software. **Xiangshu Qiu**: data curation, visualization, validation. **Ming Zhang**: conceptualization, supervision, funding acquisition, writing – original draft, writing – review and editing, data curation, project administration. **Ben Zhong Tang**: resources, conceptualization, writing – review and editing.

## Conflicts of Interest

The authors declare no conflicts of interest.

## Supporting information




**Supporting File**: advs76926‐sup‐0001‐SuppMat.docx.

## Data Availability

The datasets generated during and/or analyzed during the current study are available from the corresponding authors.
