# Supplementary material for: Overcoming Transmission Barriers: A Dual‐Functional Adhesive Hydrogel for Direct Monkeypox Virus Neutralization and Wound Healing
Source: Adv Sci (Weinh). 2026 Aug 5;13(60):e76926. doi: 10.1002/advs.76926 (PMC13440193; doi:10.1002/advs.76926)
Supplement: Supplementary file 1 [file ADVS-13-e76926-s001.docx]

*Supplementary information*

**Overcoming Transmission Barriers: A Dual-Functional Adhesive Hydrogel for Direct Monkeypox Virus Neutralization and Wound Healing**

Ming Zhang^1,+,^*, Jiaxin Tian^2,3,+^, Xiaolong Zhu^2,+^, Xiao Li^3,+^, Xiangshu Qiu^3^, Yuanyuan Li^2^, Ben Zhong Tang^5^, Shuxuan Li^4,^*, Ning Shi^2,^*

**Methods**

**Materials**

1,3,5-Trimethylbenzene (TMB), dopamine hydrochloride (DA), acrylic acid (AA), sulfobetaine methacrylate (SBMA), N, N'-methylene bisacrylamide (MBAA), ammonium persulfate (APS), N, N, N', N'-Tetramethylethylenediamine (TEMED), and acetic acid were purchased from Aladdin Biochemical Technology Company (Shanghai, China). Triblock copolymer Pluronic F127 (F127), tris(hydroxymethyl)aminomethane (TRIS), ammonia, ethanol, and acetone were from Shanghai Macklin Company (Shanghai, China). DCFH-diacetate (DCFH-DA) was purchased from Beyotime Biotechnology (Shanghai, China).

**Characterization of PDA and mPDA NPs**

The particle size and zeta potential of the prepared PDA and mPDA NPs were determined by a NICOMP 380ZLS zeta potential/particle sizer (Nano ZSP, Malvern, UK). Their surface morphology and structure were, respectively, measured using scanning electron microscopy (SEM, SU8010-HITACHI, Japan) and transmission electron microscopy (TEM, JEM-2100Plus, JEOL, Japan). The nitrogen (N_2_) adsorption/desorption isotherms and pore size distribution were analyzed by the nitrogen adsorption analyzer (Belsorp-max II, MicrotracBEL, Japan). The optical property of PDA and mPDA NPs was determined in their methanol dispersions by a double-beam UV−vis spectrophotometer (PerkinElmer Inc., MA, USA). Their molecular structure was characterized by the Fourier transform infrared spectroscopy (FTIR, 850, Tianjin Gangdong Sci. & Tech. Co., Ltd., China). The absorbance was monitored by a Microplate Reader (Multiskan Sky, Thermo Fisher Scientific, USA).

**Photothermal performance of mPDA, Polymer@mPDA, and Polymer@mPDA@Gel**

To evaluate the photothermal effects of mPDA, Polymer@mPDA, and Polymer@mPDA@Gel, 0.5 mL of mPDA aqueous solution, Polymer@mPDA aqueous solution, and Polymer@mPDA@Gel precursor solution were added to 1.5 mL centrifuge tubes, respectively, and irradiated with a 980 nm laser (1.0 W/cm^2^) for 10 minutes. To assess the photothermal effects of Polymer@mPDA under different power levels and concentrations, 0.5 mL of Polymer@mPDA aqueous solution was added to 1.5 mL centrifuge tubes and irradiated with a 980 nm laser (0.2, 0.4, 0.6, 0.8, 1.0 W/cm^2^) for 10 minutes, while temperature changes were recorded. Additionally, 0.5 mL of Polymer@mPDA aqueous solutions with different polymer concentrations (0, 25, 50, 75, 100 μg/mL) were added to 1.5 mL centrifuge tubes and irradiated with a 980 nm laser (1.0 W/cm^2^) for 10 min, and temperature changes were recorded. Furthermore, a Polymer@mPDA aqueous solution with a polymer concentration of 100 μg/mL was placed in a 1.5 mL centrifuge tube, irradiated with a 980 nm laser (1.0 W/cm^2^) for 10 minutes, and then allowed to cool in the dark for 10 min. This heating-cooling cycle was repeated for a total of 5 cycles, and the heating-cooling curves were recorded. The photothermal conversion efficiency (*η*) of PNPs was calculated using the following formula:

Where ∆*T*_max_ represents the temperature change of sample at the maximum steady-state temperature; ∆*T*_maxs_ represents the temperature change of solvent at the maximum steady-state temperature; I is the laser power density of the 980 nm light irradiation; A represents the 980 nm absorbance of Polymer@mPDA; C_s_ and m_s_ are the specific heat capacity and the total mass of H_2_O, respectively; τ is the time constant quantity of Polymer@mPDA. Hence, τ was calculated from the linear regression curve in the cooling period of Polymer@mPDA. The temperature of the solution was monitored with a PTT monitoring system MG33 (Shanghai Magnity Electronics Co., Ltd).

**Rheology assay of Polymer@mPDA@Gel**

The rheological properties of the hydrogel were tested using the frequency sweep method of a rheometer (Anton Paar MCR 302, Graz, Austria). The measurement was conducted using a 20 mm diameter fixture at 25 °C, with a test gap of 1 mm, a strain of 1%, and an angular frequency range of 0.1–100 rad/s. The measurement began after the hydrogel was applied to the testing platform. Subsequently, mineral oil was added around the testing area to seal the hydrogel and prevent dehydration.

**Mechanical properties of hydrogel**

A universal testing machine tested the mechanical properties of the hydrogel with a sensor range of 500 N. The hydrogel was formed into a cylinder (10 mm high, 8 mm diameter), and a compression test was conducted at room temperature with a compression rate of 5 mm/min and a compression strain of 80%. Stress (σ) is defined as the loading force divided by the original cross-sectional area of the specimen, and strain (ε) is defined as the deformation length divided by the original length of the specimen.

**Adhesion test of hydrogel**

Fresh pig skins were immersed in a PBS solution at room temperature, and their excess fat was removed. Then they were cut into 5 × 2 cm slices. The sheets were then re-immersed in PBS solution to ensure tissue wetting. When using the sheet, the residual PBS solution on the sheet is removed with filter paper. Then, use a spatula to evenly distribute 200 µL of the hydrogel solution on the sheet (2 × 2 cm). 200 μL of the hydrogel solution was evenly distributed on a sheet (2 × 2 cm) by a spatula, and another sheet of skin was applied to the hydrogel precursor. Then, the prepared samples were left at room temperature for 3 h. Finally, adhesion properties were tested using a lap shear test at a speed of 5 mm/min on a material testing system.

**H_2_O_2_ scavenging activity**

H_2_O_2_ scavenging capacity of mPDA, Polymer@mPDA, and Polymer@mPDA@Gel was tested by the Hydrogen Peroxide Detection Kit (Nanjing Jiancheng Bioengineering Institute, China). H_2_O_2_ reacts with ammonium molybdate to form a stable yellow complex, which displays an absorbance peak at 405 nm. Various materials of mPDA, Polymer@mPDA, and Polymer@mPDA@Gel were incubated with 2 mM H_2_O_2_ at 37 °C for 2 h, respectively. After the reaction, the concentration of remaining H_2_O_2_ was determined according to the manufacturer’s instructions, and the H_2_O_2_-scavenging capacity was calculated.

**Hydroxyl radical (·OH) scavenging activity**

The TMB chromogenic method was used to assess ·OH scavenging activity. The **·**OH was generated by the classical Fenton reaction between H_2_O_2_ and Fe^2+^, which can convert the TMB to an oxidized TMB (oxTMB) with a characteristic absorption at 652 nm. Therefore, the concentration of remaining **·**OH can be determined by monitoring the absorption at 652 nm of oxTMB. In detail, the working test solutions containing 1 mM H_2_O_2_, 1 mM FeSO_4_, and different materials of mPDA, Polymer@mPDA, and Polymer@mPDA@Gel in HAc/NaAc buffer (0.5 M, pH 4.5) were prepared in the dark and rested for 10 min. Then TMB was added to each group and fully shaken. Afterward, the absorbance peak at 652 nm of the solution was monitored with a multiple plate reader.

**Superoxide anion (·O_2_^−^ ) scavenging activity**

The ·O_2_^−^ scavenging activity was assessed using a superoxide anion assay kit (Nanjing Jiancheng Bioengineering Institute, Nanjing, China) according to the manufacturer’s instructions. Briefly, ·O_2_^−^ was generated through the oxidation of xanthine by xanthine oxidase (XO), which can convert the WST-1 into WST-1 formazan with a characteristic absorption at 450 nm. Different materials of mPDA, Polymer@mPDA, and Polymer@mPDA@Gel were added to the working solution. The formazan concentration was determined at 450 nm using a multiple plate reader after standing for 20 min.

**ABTS radical scavenging activity**

The evaluation of ABTS radical scavenging activity was assessed using a total antioxidant capacity test kit (Nanjing Jiancheng Bioengineering Institute, Nanjing, China) according to the manufacturer’s instructions. Various materials of mPDA, Polymer@mPDA, and Polymer@mPDA@Gel were incubated with the reagent and ABTS at 37 °C for 6 min, respectively. Afterward, the absorbance peak at 405 nm was monitored with a multiple plate reader. The ABTS radical scavenging abilities were calculated as follows:

*ABTS scavenging ratio* (%) =$\text{ }\frac{\text{A}_{\text{control}}\text{ -}\text{A}_{\text{sample}}}{\text{A}_{\text{control}}}$ × 100%

Where *A_control_* is the absorbance of a standard without any radical scavengers, and *A_sample_* is the absorbance after the reaction with the radical scavengers, respectively.

**Superoxide dismutase (SOD)-like activity**

The SOD-like activity of mPDA, Polymer@mPDA, and Polymer@mPDA@Gel was determined by formazan formation using a SOD assay kit (WST-1 method) (Nanjing Jiancheng Bioengineering Institute, Nanjing, China). Briefly, O_2_^−^ was generated through the oxidation of xanthine by XO, which can convert the WST-1 into WST-1 formazan with a characteristic absorption at 450 nm. The formazan concentration was determined at 450 nm using a multiple plate reader.

**Glutathione peroxidase (GPx)-like activity**

The consumption of GSH was detected with a DTNB probe. 1 mL of different concentrations of mPDA, Polymer@mPDA, and Polymer@mPDA@Gel solutions (0, 50, 100, 150, and 200 µg/mL) was mixed with 1 mL of GSH (250 μM) at room temperature. After 30 minutes of co-incubation, 100 μL of DTNB in PBS (500 μM) was added to the mixture. The absorbance of this mixed solution was then recorded using UV-Vis spectroscopy.

**Catalase (CAT)-like activity**

The CAT-like activity and the steady-state kinetics were assayed using fluorescence spectroscopy. Typically, terephthalic acid (TPA) is a non-fluorescent compound, which can react with ·OH, decomposed from H_2_O_2_, to produce a fluorescent aromatic hydroxylated product (2-hydroxyterephthalic acid) with excitation/emission peaks at 315/425 nm, respectively. In the presence of CAT or CAT analogs, the H_2_O_2 that_ decomposed into H_2_O and O_2_ could not produce the fluorescent 2-hydroxyterephthalic acid. Hence, the CAT-like activity of the Polymer@mPDA@Gel was investigated by monitoring the fluorescence signal of the system. The steady-state kinetic assay of Polymer@mPDA@Gel was performed to confirm the enzymatic catalysis mechanism by varying the concentration of H_2_O_2_ (5, 10, 20, 40, 80, and 120 mM) in the presence of Polymer@mPDA@Gel (200 µg/mL). Firstly, 200 μL 1000 µg/mL of Polymer@mPDA@Gel solution was added into the PBS (10 mM, pH 7.4), different concentrations of H_2_O_2_ were added and incubated at 40 °C for 4 h. Finally, the TPA in 2 mM NaOH was added and the fluorescence spectra of the resultant solution were recorded under an excitation of 315 nm, and the variation of fluorescence emission at 425 nm was adopted to quantify remaining H_2_O_2_. Apparent kinetic parameters were calculated based on the Michaelis–Menten equation.

$$\text{A}\text{=}\text{E}\text{bc}$$

$$\text{v}_{\text{o}}\text{=}\frac{\text{v}_{\text{max}}\text{⋅}\left[ \text{s} \right]}{\text{k}_{\text{m}}\text{+}\left[ \text{s} \right]}$$

$$\frac{\text{1}}{\text{v}_{\text{0}}}\text{=}\frac{\text{k}_{\text{m}}}{\text{v}_{\text{max}}}\text{⋅}\frac{\text{1}}{\text{s}}\text{+}\frac{\text{1}}{\text{v}_{\text{max}}}$$

**Intracellular ROS scavenging activity**

2′,7′-Dichlorofluorescin diacetate (DCFH-DA), an oxidation-sensitive fluorescent dye, was used to detect the intracellular ROS level. Briefly, DCFH-DA is a nonfluorescent chemical compound that can diffuse through cell membranes freely and can be hydrolyzed by intracellular esterase to DCFH. The non-fluorescent DCFH could be oxidized by the intracellular ROS to fluorescent DCF. Therefore, the quantity of intracellular ROS is correlated with the fluorescence intensity of DCF. Mouse macrophage cells (RAW 264.7) were cultured in DMEM medium containing 10% fetal bovine serum and incubated at 37 °C in a 5% CO_2_ incubator until they reached the logarithmic growth phase. The cells were seeded into a 96-well plate at a density of 5×10^4^ cells per well and cultured for 24 h. After incubation, the cells were treated with fresh medium, lipopolysaccharide (LPS), LPS + mPDA, LPS + polymer@mPDA, and LPS + polymer@mPDA@Gel, respectively, and co-cultured for another 24 h. Following treatment, the medium in each well was discarded, and the cells were gently washed three times with PBS. Then, 100 μL of DCFH-DA working solution was added to each well, and the cells were incubated at 37 °C in a 5% CO_2_ incubator in the dark for 30 min. After incubation, the cells were washed three times with PBS to remove any residual solution, and imaging was performed under a fluorescence microscope.

**Hemolysis assay**

The hemocompatibility of Polymer@mPDA@Gel was evaluated via a hemolysis assay. Fresh anticoagulated rabbit blood (2 mL) was centrifuged at 3000 rpm for 10 minutes to collect red blood cells (RBCs), which were washed three times with normal saline and resuspended in saline at a 1:24 (v/v) dilution. Gel or Polymer@mPDA@Gel was mixed with the diluted RBC suspension at a 1:1 volume ratio and incubated at 37 °C for 3 hours. After centrifugation at 1500 rpm, the supernatant was transferred to a 96-well plate, and absorbance was measured at 545 nm. RBC morphology was examined microscopically. Negative and positive controls consisted of RBCs mixed with saline and deionized water, respectively. The hemolysis rate was calculated as follows:

*Hemolysis rate = (OD_s_−OD_nc_) / (OD_pc_−OD_nc_)* *×100%*

where O*D_s_*, O*D_nc_*, and O*D_pc_* represent the absorbance of the sample, negative control, and positive control, respectively.

**Cell viability under viral challenge**

Cell viability under MPXV infection was assessed using the Cell Counting Kit-8 (CCK-8, Dojindo). Vero E6 cells were seeded in 96-well plates (1 × 10⁴ cells/well) and cultured overnight. A viral suspension (10⁵ TCID₅₀ in 200 µL DMEM) was pre-incubated with PBS, Gel, or Polymer@mPDA@Gel for 30 minutes, with or without subsequent 980 nm laser irradiation (0.5 W cm⁻², 5 minutes). After viral adsorption, cells were washed and maintained in DMEM with 1% FBS and antibiotics for 12 hours. Cells were then incubated with CCK-8 solution (10% v/v) for 1 hour, and absorbance was measured at 450 nm. Cytotoxicity was calculated as:

*Cytotoxicity (%)  = (Abs._sample_  −  Abs._blank_) / (Abs._negative control_  −  Abs._blank_) ×  100*

where *Abs._sample_*, *Abs._blank_*, and *Abs._negative control_* represent the absorbance of the sample, negative control, and positive control, respectively.

**Virus titration by TCID_50_ assay**

Vero E6 monolayers in 96-well plates were inoculated with 10-fold serial dilutions of virus and incubated at 37 °C for 4 days. Viral titers, expressed as 50% tissue culture infectious dose (TCID*_50_*), were determined in octuplicate and calculated using the Reed and Muench method.

**Quantitative reverse transcription PCR (qRT-PCR)**

Total RNA was extracted using TRIzol reagent (Invitrogen) and reverse-transcribed using random or oligo(dT) primers with the Transcriptor First Strand cDNA Synthesis Kit (Roche). qRT-PCR was performed in triplicate using FastStart Essential DNA Green Master (Roche) on a LightCycler® system. Gene expression levels of IL-10, TGF-β, TNF-α, IFN-g, and IL-1β were normalized to GAPDH and analyzed via the 2^(–ΔΔCt) method.

**Histopathology and immunohistochemistry**

Skin and leg tissue specimens were fixed in 4% paraformaldehyde for 14 days, embedded in paraffin, sectioned, and stained with hematoxylin and eosin (H&E) according to standard protocols. Images were acquired using a light microscope.

**Cytokine measurement by ELISA**

The enzyme immunoassay kits for IL-10 (SEA056Mu, SEA056Rb), IFN-g (SEA049Mu, SEA049Rb), TNF-α (SEA133Mu, SEA133Rb, SEA133Si) were obtained from Cloud-Clone (Wuhan, China), and TGF-β (SEKM-0035, SEKRT-0401) was obtained from Solarbio (Beijing, China). Experiments were carried out according to the manufacturer’s instructions.

**RNA Sequencing**

Total RNA from cynomolgus macaque skin was extracted with TRIzol, and RNA-seq libraries were constructed and sequenced on the Illumina HiSeq6000 platform (2 × 150 bp). Clean reads were aligned to the Macaca fascicularis genome (GCF_000364345.1) using HISAT2. Gene expression levels were quantified as FPKM using featureCounts. Differentially expressed genes (DEGs) were defined as those with |log₂(fold change)| ≥ 1 and P ≤ 0.05. Functional enrichment analysis of GO terms and KEGG pathways was conducted using clusterProfiler. Heatmaps were generated with the pheatmap package in R, and protein–protein interaction networks were analyzed via STRING.

**Statistical analysis**

All quantitative data are presented as mean ± standard deviation (s.d.) from at least three independent experiments. Differences between the two groups were assessed using two-tailed unpaired Student’s t-tests, while multiple comparisons were analyzed by one-way ANOVA with post-hoc correction. A P-value < 0.05 was considered statistically significant. All analyses were performed using GraphPad Prism 5.

**Supplementary figures**

**Supplementary Figure 1.** The synthetic route of the polymer.

**Supplementary Figure 2.** SEM images and particle size histograms of PDA. Scale bar, 200nm.

**Supplementary Figure 3.** Thermal images of mPDA and polymer@mPDA.

**Supplementary Figure 4.** Thermal images of Polymer@mPDA under different power levels.

**Supplementary Figure 5.** Thermal images of Polymer@mPDA with different polymer concentrations.

**Supplementary Figure 6.** Heating/cooling images of Polymer@mPDA.

**Supplementary Figure 7.** (A) Kinetic viscosity tests to evaluate the flow properties of Polymer@mPDA@Gel. (B, C) Shear stress tests to determine the response of Polymer@mPDA@Gel to different shear rates.

**Supplementary Figure 8.** (A) Schematic illustration of the compression experiment. (B) Compression properties of the Gel and Polymer@mPDA@Gel. (C) Photographs of Polymer@mPDA@Gel subjected to bending, twisting, and stretching.

**Supplementary Figure 9.** (A) Schematic diagram of the shear experiment. (B) Shear adhesive strength of the Gel and Polymer@mPDA@Gel.

**Supplementary Figure 10.** Adhesive properties of Polymer@mPDA@Gel to various substrates.

**Supplementary Figure 11.** Thermal images of mPDA, polymer@mPDA, and Polymer@mPDA@Gel.

**Supplementary Figure 12.** Heating/cooling images of Polymer@mPDA@Gel.

**Supplementary Figure 13.** (A) Michaelis-Menten kinetic analysis and (B) Lineweaver-Burk plotting for polymer@mPDA@Gel with H_2_O_2_ as a substrate.

**Supplementary Figure 14.** Hemolysis images of RBCs after incubation with mPDA, Polymer@mPDA, and Polymer@mPDA@Gel.

**Supplementary Figure 15.** HE staining of major organs 21 days after intraperitoneal injection of Polymer@mPDA@gel.

**Supplementary Figure 16.** Cytokine (IL-10, TGF-β, TNF-α, and IFN-g) levels in reinoculated rabbits via ELISA. Data are presented as mean ± s.d. (n = 4). One-way ANOVA was used to determine significance, with post-hoc testing.
